# The role of HGF-MET pathway and CCDC66 cirRNA expression in EGFR resistance and epithelial-to-mesenchymal transition of lung adenocarcinoma cells

**DOI:** 10.1186/s13045-018-0557-9

**Published:** 2018-05-31

**Authors:** Nithila A. Joseph, Shiow-Her Chiou, Zoe Lung, Cheng-Lin Yang, Tze-Yi Lin, Hui-Wen Chang, H. Sunny Sun, Sachin Kumar Gupta, Laising Yen, Shulhn-Der Wang, Kuan-Chih Chow

**Affiliations:** 1 Graduate Institute of Biomedical Sciences, National Chung Hsing University, Taichung, Taiwan; 2 Graduate Institute of Microbiology and Public Health, National Chung Hsing University, Taichung, Taiwan; 3 Department of Molecular and Cell Biology, University of California, Berkeley, CA USA; 4 Department of Pathology, China Medical University Hospital, Taichung, Taiwan; 5 Institute of Molecular Medicine, National Chung Kung University, Tainan, Taiwan; 6 Department of Pathology and Immunology, Baylor College of Medicine, Houston, TX USA; 7 School of Post-Baccalaureate Chinese Medicine, College of Chinese Medicine, China Medical University, Taichung, Taiwan

**Keywords:** SUMOylation, EGFR, SAE2, EMT, HGF, c-MET, cirRNA, CCDC66

## Abstract

**Background:**

Epithelial-to-mesenchymal transition (EMT) has, in recent years, emerged as an important tumor cell behavior associated with high metastatic potential and drug resistance. Interestingly, protein SUMOylation and hepatocyte growth factor could respectively reduce the effect of small molecule inhibitors on tyrosine kinase activity of mutated epidermal growth factor receptor of lung adenocarcinomas (LADC). The actual mechanism is yet to be resolved.

**Methods:**

Immunohistochemistry was used to stain proteins in LADC specimens. Protein expression was confirmed by Western blotting. In vitro, expression of proteins was determined by Western blotting and immunocytochemistry. Levels of circular RNA were determined by reverse transcription-polymerase chain reaction.

**Results:**

SAE2 and cirRNA CCDC66 were highly expressed in LADC. Expression of SAE2 was mainly regulated by EGFR; however, expression of cirRNA CCDC66 was positively regulated by FAK and c-Met but negatively modulated by nAchR7α. EGFR-resistant H1975 also highly expressed cirRNA CCDC66. Immediate response of hypoxia increased phosphorylated c-Met, SAE2, and epithelial-to-mesenchymal transition. Either activation of FAK or silencing of nAchR7α increased cirRNA CCDC66.

**Conclusions:**

HGF/c-Met regulates expression of SAE2 and cirRNA CCDC66 to increase EMT and drug resistance of LADC cells. Multimodality drugs concurrently aiming at these targets would probably provide more benefits for cancer patients.

**Electronic supplementary material:**

The online version of this article (10.1186/s13045-018-0557-9) contains supplementary material, which is available to authorized users.

## Background

Elevated tumor cell proliferation and metastatic potential are major characteristics of lung adenocarcinoma (LADC), associating with increased resistance to radio- and chemotherapy, as well as early tumor recurrence, fast disease progression, and high mortality rate [1, 2]. Although public policy of restricting cigarette retails and smoking has improved living environments, the annual incidence and death of lung cancer increase incessantly, including the USA and Taiwan [3] (https://www.mohw.gov.tw/lp-3327-2.html). The appalling escalation was particularly notable in female patients. Unfortunately, many new patients were mostly young non-smokers who died of lung cancer due to the early recurrence and multi-organ involvement [1–3] (http://www.mohw.gov.tw/cht/DOS/Statistic.aspx).

Recent development of chemotherapy targeting mutated human epidermal growth factor receptor (EGFR) by tyrosine kinase inhibitors (TKIs) has become a major strategic treatment for patients in the Far East [4]. However, not all patients with the same clinical features had the same treatment achievements [5]. Moreover, TKI improved only progression-free survival but not overall survival, suggesting that TKIs might not be effective for full-blown and large-scale metastasis and that sensitivity of tumor cells might be affected by other factors. To determine whether the disease progression correlates with specific gene expression profiles, we combined differential displays and microarrays to analyze gene expression patterns in LADC specimens at various stages of disease progression [6]. We identified several potential genes, including aldo-keto reductases (also named dihydrodiol dehydrogenase, DDH), hepatocyte growth factor (HGF), HGF receptor (or c-Met), dynamin-related protein 1 (DRP1) and the ATPase family, and AAA domain-containing 3A (ATAD3A) [6–9]. Interestingly, the protein level of ATAD3A increased dramatically when cancer cells were serum-starved. Furthermore, through nicotinic acetylcholine receptor alpha 7 (nAChRα7), nicotine could increase expression of HGF and eukaryotic elongation factor 2 (eEF2), which in turn upregulate ATAD3A and DRP1 [6, 10]. Nicotine also increases eEF2 SUMOylation, which is vital for maintaining anti-apoptotic capability of LADC.

SUMOylation is a post-translational modification of proteins by a SUMO (small ubiquitin-related modifier) protein (conjugating to an epsilon lysine residue of the substrate protein) and induces protein conformation change to enhance transcription and cell cycle progression [11]. Before reaction, the C-terminus of SUMO precursor is removed by SUMO-specific proteases (SUPs) to expose the glycine-glycine motif, which is activated by a heterodimeric enzyme complex, composed of SUMO-activating enzyme subunit 1 (SAE1) and SAE2, with the expense of an ATP [12]. The activated SUMO is transferred from SAE1/2 to an E2 conjugating protein, ubiquitin-conjugating (UBC) 9, and subsequently conjugated to the final substrates by an E3 ligase, e.g., SP-RING-type (Siz/PIAS-family) E3 ligase or IR-type (internal repeats) E3 ligase [11]. Four types of SUMO proteins have been identified; however, they are all activated by the same SAE1/2 and transferred to the substrate proteins via UBC9 [11–14].

Using a genome-wide RNA interference method to screen cancer cell growth-related genes, Kessler et al. found that expression of SAE1/2 was correlated with higher metastatic potential of cancer cells [15]. SUMOylation of BrCa1 has been shown to increase DNA damage repair and drug resistance [16]. Moreover, SUMOylation of SIRT1, a class III deacetylase sirtuin-1, was found to drive epithelial-to-mesenchymal transition (EMT) [17]. Interestingly, SUMOylation of GTPase Rac1, which was activated by HGF and PI3K/Akt, not only increased cell migration [18], but also reduced effectiveness of gefitinib on EGFR mutants [19]. A recent study by Du et al. showed that SAE2 increased Oct-1 stability to maintain stemness of cancer cells [20]. However, the relationships among SAE2, ATAD3A, EGFR, HGF, and EMT are yet to be determined. In this report, we showed their correlations via coiled-coil domain containing 66 (CCDC66) circular RNA (cirRNA) to induce EGFR resistance as well as to increase EMT and metastatic potential of LADC cells.

## Results

### Expressions of intracellular markers, ATAD3A and SAE2, influence the sensitivity of EGFR-mutated LADC cells to tyrosine kinase inhibitors

**Fig. 1:**
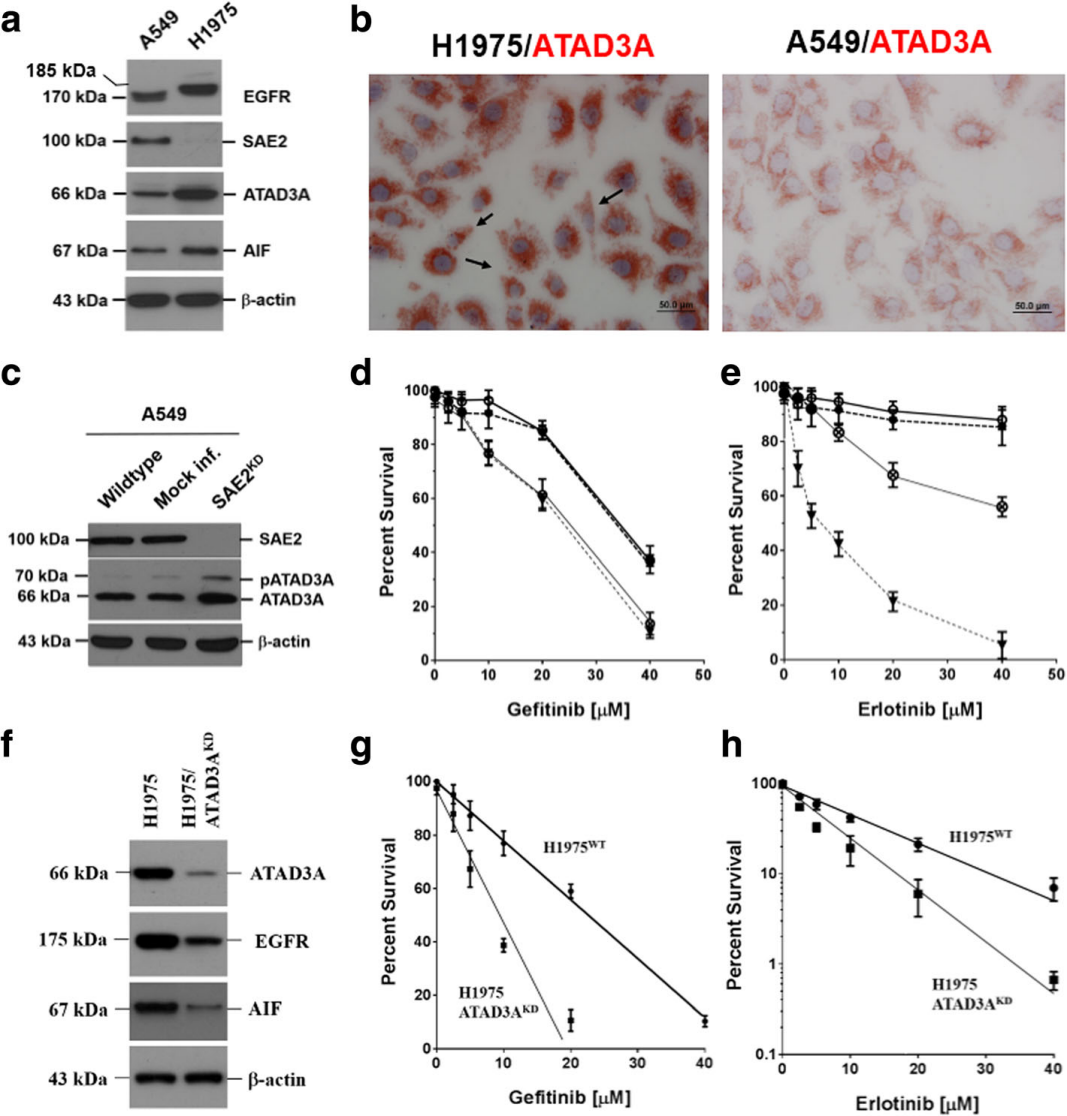
Expression of intracellular markers, SAE2 and ATAD3A, affect sensitivity of EGFR-mutated lung adenocarcinoma (LADC) cells to tyrosine kinase inhibitors, gefitinib or erlotinib. **a** LADC cells, carrying mutated EGFR (H1975), expressed abundant ATAD3A, an anti-apoptotic factor [9], as determined by a Western blotting analysis. **b** ATAD3A protein was highly expressed in EGFR-mutated H1975 (the left panel) and, intermediately, in the wild-type A549 (the right panel) cells. Some of the EGFR-mutated H1975 cells were elongated, scattered, and crescent shape (dark arrows), resembling phenotypes of mesenchymal cells [21], implicating that mutated EGFR could be associated with epithelial-to-mesenchymal transition (EMT) of LADC cells. **c** Knockdown of SAE2 expression (SAE2 ) increased ATAD3A expression. **d** In SAE2^KD^ cells, gefitinib sensitivity was also increased, suggesting that SAE2 could be an intracellular sensor of gefitinib. It is therefore worth noting that gefitinib sensitivity of SAE2^KD^ A549 cells was equivalent to that of H1975 cells (ID50 ~ 25 μM). **e** Silencing of SAE2 expression increased erlotinib sensitivity of A549 cells as well. However, SAE2^KD^ A549 cells (ID ~ 50 μM) were more resistant than H1975 cells (ID ~ 5.6 μM) to erlotinib. **f** Silencing of ATAD3A (ATAD3A ) reduced expression of EGFR and AIF. **g** Increase of gefitinib sensitivity was thus anticipated in ATAD3A^KD^ cells. **h** Increment of erlotinib sensitivity was also noted in ATAD3A^KD^ cells

LADC cells with mutant EGFR (H1975 cells) overexpressed ATAD3A (Fig. 1a, b), an anti-apoptotic factor [9], but rarely expressed SAE2. Molecular weight of EGFR in H1975 (~ 185 kDa) was higher than in A549 (170 kDa), suggesting that the protein was phosphorylated. Interestingly, some of H1975 cells (dark arrows) resembled phenotypes of mesenchymal cells [21] (Fig. 1b). Silencing of SAE2 increased ATAD3A expression (Fig. 1c) and sensitivity of A549 cells to gefitinib (Fig. 1d) and erlotinib (Fig. 1e). Sensitivity of SAE2^KD^ A549 cells to gefitinib was equivalent to that of H1975 cells (ID_50_ ~ 25 μM). However, SAE2^KD^ A549 cells (ID_50_ ~ 50 μM) were more resistant than H1975 cells (ID_50_ ~ 5.6 μM) to erlotinib. Silencing of ATAD3A (ATAD3A^KD^), on the other hand, reduced expression of EGFR and AIF (Fig. 1f), however, increased sensitivity to both (Fig. 1g) gefitinib and (Fig. 1h) erlotinib.

### Expression pattern of membrane receptors and intracellular transport markers in LADC cell lines

**Fig. 2:**
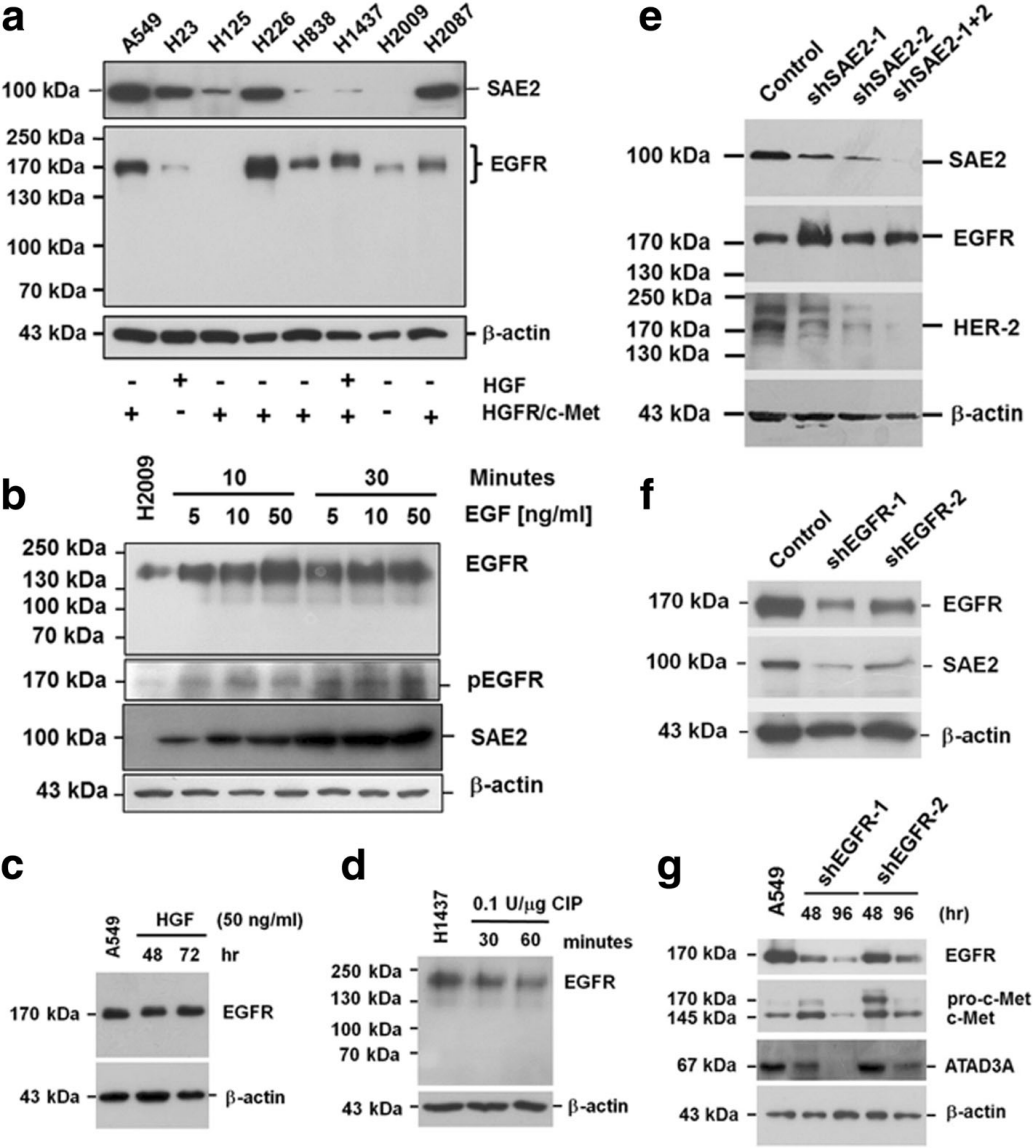
Expression pattern of membrane receptors and intracellular transport markers in lung cancer cell lines. **a** Expression of EGFR detected by immunoblotting was high in A549, H226, and H1437; moderate in H838 and H2087; and low in H23 and H2009 cells. EGFR was not detected in H125 cells. Molecular weight of EGFR in H838, H2087 (about 175 kDa), and H1437 (about 185 kDa) was higher than that in A549, H23, H226, and H2009 (about 170 kDa) cells. Interestingly, only H1437 cells expressed both HGF and c-Met. Expression of SAE2, on the other hand, was detected as a 100-kDa protein in lung cancer cells. Expression of β-actin was a monitoring standard. SAE2 expression was high in A549, H23, H226, and H2087; moderate in H125; and low in H838 and H1437 cells. SAE2 was not detected in H2009 cells. **b** Addition of EGF increased expression level and the phosphorylated forms (pEGFR) of EGFR in H2009 cells, which did not express c-Met. However, no evident molecular weight upshifting of EGFR was detected. Addition of EGF increased SAE2 protein expression as well. **c** Addition of HGF increased molecular weight upshifting of EGFR from 170 to 185 kDa in A549 cells. **d** Following treatment of H1437 cells with calf intestinal phosphatase (CIP), protein levels and molecular weights of 185 kDa EGFR reduced, suggesting that phosphorylation was critical for EGFR stability. **e** Using siRNA to knockdown EGFR (EGFR^KD^) expression for 48 h reduced protein levels of SAE2. **f** Silencing of EGFR gene expression for 48 h (EGFR^KD^-48hr) reduced SAE2 expression. g However, expression of c-Met and pc-Met was increased in EGFR^KD^-48hr cells. Such short-term effect dwindled when EGFR gene silencing was continued to 96 h; and at this time, protein level of c-Met and ATAD3A reduced markedly

In vitro, expression of EGFR was low in H23 and H2009, moderate in H838 and H2087 (5- to 10-fold higher than in H23 and H2009 cells), and high in A549, H226, and H1437 cells (10- to 30-fold higher than in H23 and H2009 cells) (Fig. 2a, the upper panel). H1437 cells that had a molecular weight upshift was also positive for c-Met and HGF. Expression of SAE2 was low in H838 and H1437, moderate in H125 (5- to 10-fold higher than in H838 and H1437 cells), and high in A549, H23, H226, and H2087 cells (20- to 40-fold higher than in H838 and H1437 cells) (Fig. 2a, the lower panel). Interestingly, molecular weights of EGFR in H838, H2087 (~ 175 kDa), and H1437 (~ 185 kDa) were higher than those in A549, H23, H226, and H2009 (~ 170 kDa) cells. Moreover, sensitivity of H1437 to gefitinib was equivalent to that of H1975 [22].

Although addition of EGF increased levels of phosphorylated EGFR (pEGFR), no obvious molecular weight upshift of EGFR was observed (Fig. 2b). Addition of hepatocyte growth factor (HGF), on the other hand, increased molecular weight upshift of EGFR (from 170 to 185 kDa) (Fig. 2c). Treatment with 0.1 U/μg of calf intestinal phosphatase (CIP) downshifted EGFR molecular weight in H1437 cells (Fig. 2d). Silencing of SAE2 did not evidently affect protein level of EGFR but reduced HER2 levels (Fig. 2e). Silencing of EGFR reduced protein level of EGFR and that of SAE2 (Fig. 2f). Short-term silencing of EGFR increased levels of c-Met and pro-c-Met. Prolonged silencing of EGFR for over 96 h, on the other hand, diminished both c-Met and ATAD3A (Fig. 2g), suggesting a physiological feedback circuit for regulating homeostasis of intracellular material transport system, which could then sustain cell survival under conditions of growth factor deficiency or with defected receptors [6–10].

### Biological correlations of EGFR with drug resistance, epithelial-to-mesenchymal transition, and metastatic potential of LADC cells

**Fig. 3:**
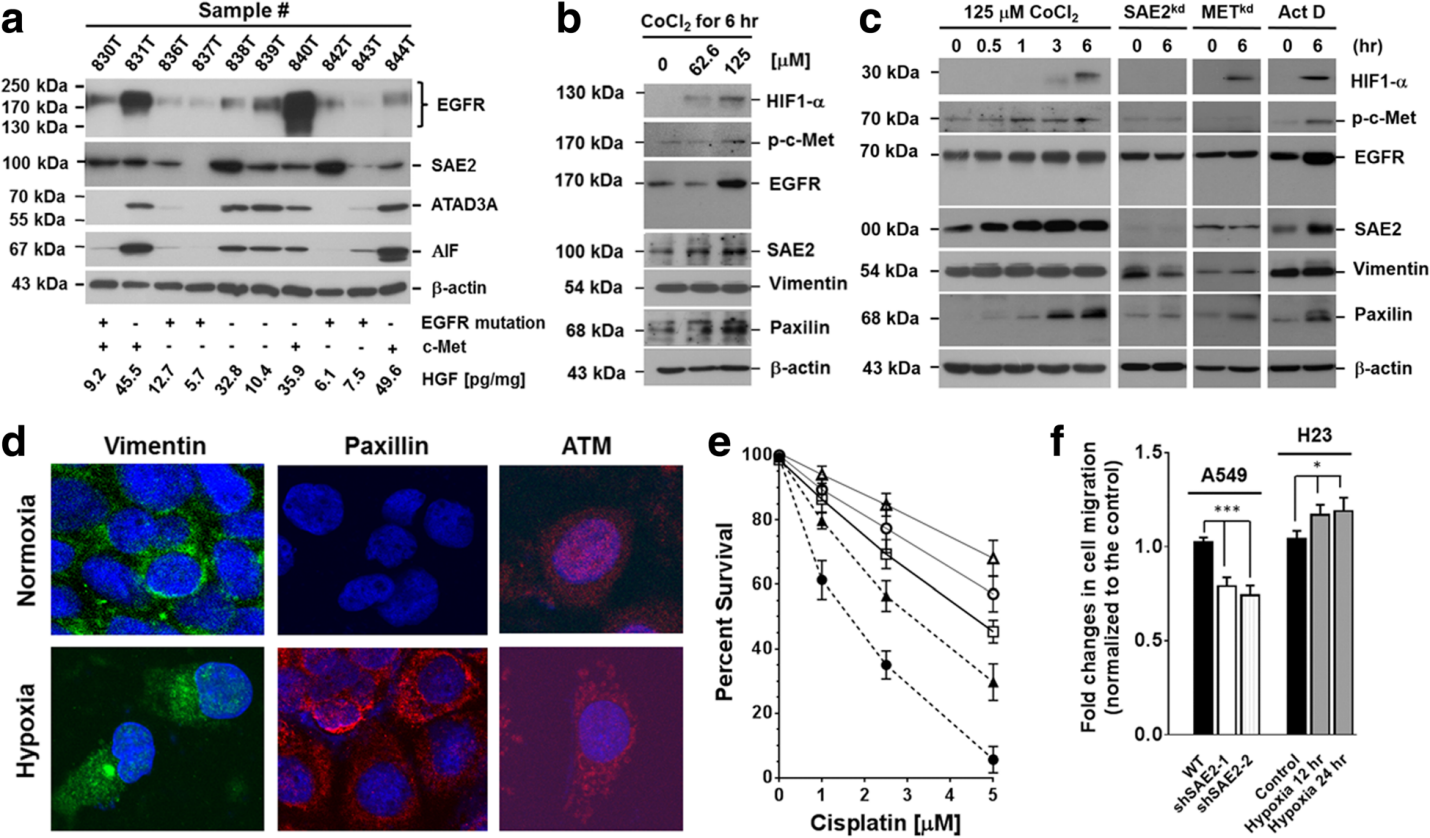
Biological correlations of EGFR with drug resistance, epithelial-to-mesenchymal transition, and metastatic potential of LADC cells. **a** In vitro, we had shown that the different molecular weights of EGFR in various NSCLC cell lines were closely associated with HGF and c-Met pathways. Molecular weights of EGFR in surgical biopsies of LADC also varied from patient to patient (tumor samples 830, 831, 836, 840, and 844 above 170 kDa; tumor specimens 839, 842, and 843 around 170 kDa). Interestingly, protein levels of mutant EGFR were generally lower than those of wild type. Samples that had higher molecular weights were mostly positive for c-Met and higher serum HGF, supporting our in vitro results that EGFR could be modified through HGF/c-Met pathway. **b** Using CoCl_2_ to induce hypoxia-activated c-Met phosphorylation (p-c-Met) increased protein level of EGFR and SAE2, as well as epithelial-to-mesenchymal transition (EMT) markers, e.g., vimentin and paxillin, in a dose-dependent manner in H23 cells. **c** c-Met phosphorylation and increase of the related proteins were initiated about 30–60 min following CoCl_2_ treatment of H23 cells. Appearance of HIF-1α became visible about 3–6 h following CoCl_2_ challenge, indicating that the increase of EMT-related protein expression occurred before traditional hypoxic activation. The hypoxia-mediated protein increases were abolished when SAE2 gene was silenced. Moreover, addition of actinomycin D did not affect CoCl_2_-induced cell responses. **d** In addition to the increases of proteins as shown by Western blotting analysis, confocal immunofluorescence micrographs revealed that hypoxia altered protein distribution inside the cells as well. For instance, vimentin was accumulated in the enlarged cytoplasmic vacuole-like structures, but not on the plasma membrane. Intracellular distribution of paxillin was generally similar to that of vimentin, except that some cells started to have nuclear signals of paxillin. Nuclear signals of ATM, however, were markedly reduced, and most of the proteins were retained in the cytoplasm in elongated spherical forms. **e** Exposure of H23 cells (□, control) to hypoxic condition increased resistance to cisplatin (○, hypoxia for 12 h; △, hypoxia for 24 h). Using shRNA-lentivirus to silence SAE2 gene expression (by shSAE2-1 [▲] or by shSAE2-2 [●]) increased sensitivity of LADC cells to cisplatin. **f** Silencing of SAE2 expression in A549 cells reduced cell mobility across extracellular matrix protein-coated carbonate membrane (the left panel). When H23 cells were exposed to hypoxia prior to the cell mobility examination, the cell migration ability increased significantly (the right panel). The statistical differences were significant (WT A549 vs. shSAE2-1 and shSAE2-2, *P* < 0.0004 [***], ANOVA; control H23 vs. H23 cells exposed to hypoxia, *P* < 0.0358 [*], ANOVA). [*P < 0.05; ***P < 0.001]

In patient’s biopsies, molecular weights of EGFR varied from patient to patient (tumor specimens 830, 831, 836, 837, 840, and 844 above 170 kDa; tumor specimens 839, 842, and 843 about 170 kDa) (Fig. 3a). Protein level of mutant EGFR was in general lower than that of the wild type. Samples having EGFR molecular weight upshifting were mostly positive for c-Met, supporting our previous results that HGF/c-Met system was critical for the function of EGFR. While SAE2 expression correlated with EGFR, expression of ATAD3A and AIF was markedly reduced in LADC samples with mutant EGFR.

Previously, we had shown that c-Met was highly expressed in LADC biopsies and cell lines [6]. Patients with smoking habits usually had higher HGF level in the diseased lungs. In vitro, hypoxia increased HGF level and c-Met phosphorylation. Using CoCl_2_ to deplete intracellular oxygen also increased phosphorylated c-Met and levels of EGFR and SAE2, as well as markers of epithelial-to-mesenchymal transition (EMT), e.g., vimentin and paxillin, in LADC cells. The increase was dose- and time-dependent (Fig. 3b, c). Silencing of SAE2 or c-MET alleviated the effect of hypoxia on EMT markers’ expression (Fig. 3c). Besides protein levels (as measured by Western blotting analysis), hypoxia altered intracellular protein distribution (Fig. 3d, as shown by confocal immunofluorescence microscopy). Vimentins were gathered in cytoplasmic vacuole-like structures. In this way, vimentins on plasma membranes were markedly reduced. Hypoxia affected intracellular distribution of paxillin and nuclear ataxia-telangiectasia-mutated (ATM) kinase as well. Expression of ATM was clearly reduced, and most of the proteins were retained mostly in the cytoplasm as irregularly orbicular vesicles, suggesting that hypoxia could interfere intracellular material transport, which fortuitously activated cell survival response [6–10, 23].

Exposure of LADC cells to CoCl_2_ for 12–24 h increased SAE2 expression and cisplatin resistance (Fig. 3e). Silencing of SAE2 expression reduced not only cisplatin resistance (Fig. 3e), but also cell mobility across Matrigel-coated membrane (Fig. 3f). Hypoxia increased cell mobility. The difference was statistically significant (WT vs. shSAE2-1- or shSAE2-2-treated A549 cells, *P* < 0.0004, ANOVA; control H23 vs. hypoxic H23 cells, *P* <  0.0358, ANOVA).

### Pathologically, EGFR and SAE2 were concurrently expressed in LADC patients with poor prognosis

**Fig. 4:**
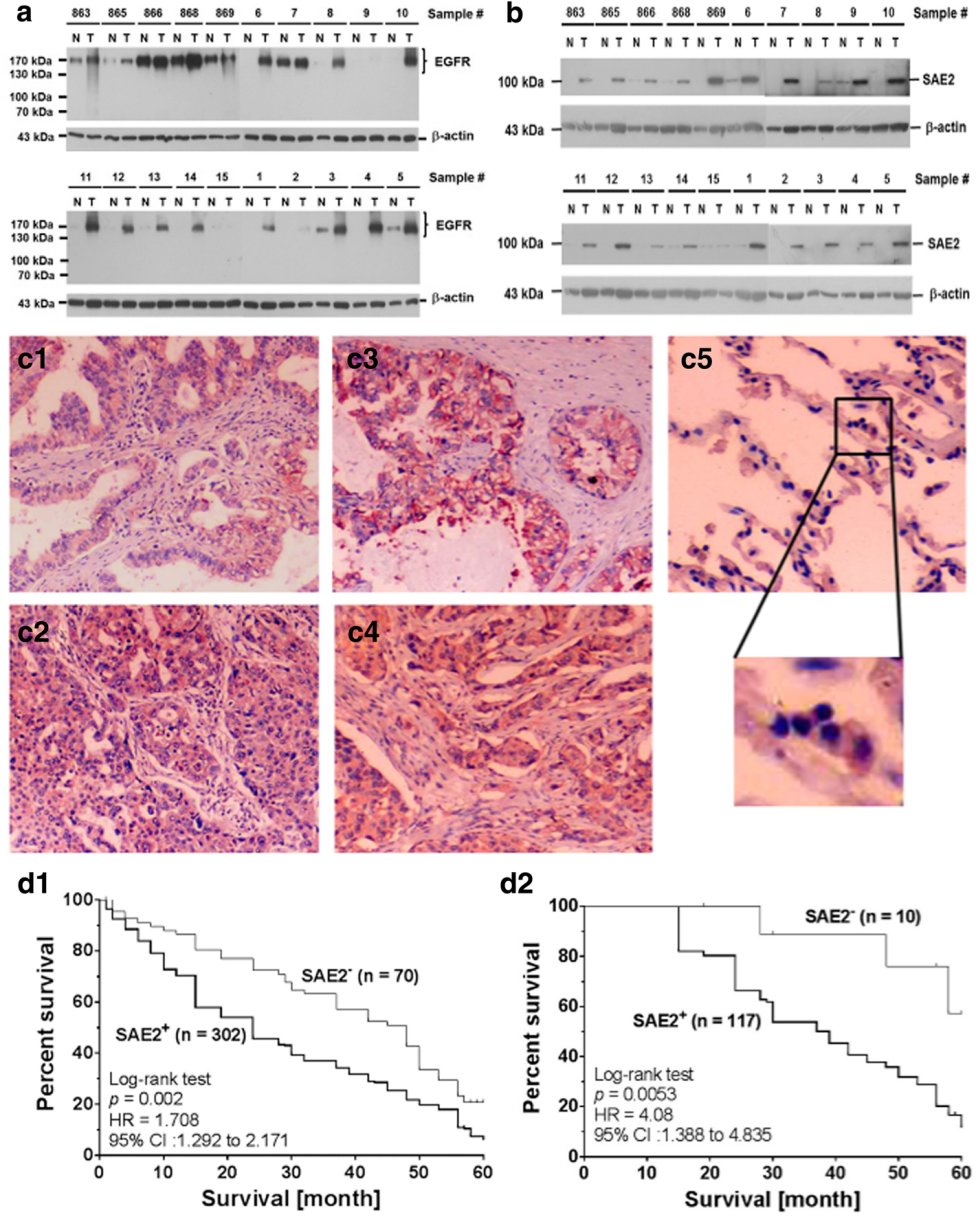
Pathologically, EGFR and SAE2 were concurrently expressed in LADC patients with poor survival. **a** Using a Western blotting method, expression of EGFR was detected with various molecular weights in 18 tumor and eight non-tumor fractions of 20 pair of LADC biopsies. β-actin was used as a monitoring standard. **b** Using the same method, expression of SAE2 was detected as a 100-kDa protein in 19 tumor and two non-tumor fractions of LADC specimens. N non-tumor lung tissue (NTLT), T tumor fraction of surgical resections. **c** Representative examples of SAE2 expression in pathological specimens of LADC was determined by immunohistochemical staining (crimson precipitates). Expression of SAE2 was detected in the LADC tumor nests of (c1) papillary, (c2) acinar, (c3) solid, and (c4) mixed subtypes. (c5) In the NTLT, signal of SAE2 was detected in type II pneumocytes (the enlarged area), but not in type I pneumocytes of the air sac. **d** Comparison of Kaplan-Meier product limit estimates of survival analysis in LADC patients. (d1) Patients were divided into two groups depending on the expression of SAE2. The difference in survival between the two groups was compared by the log-rank test. Survival of patients with low SAE2 level was significantly better than those with high SAE2 level. The hazard ratio between these two groups was 1.708, and the difference in cumulative survival was significant (*P* = 0.002). (d2) Survival of stage I patients with low SAE2 level was significantly better as well. The hazard ratio was 4.08, and the difference in cumulative survival was significant (*P* = 0.0053)

Expression of EGFR and SAE2 was determined by Western blotting in 66 pairs of NTLT and LADC specimens. Both EGFR (84.8%, 56/66) and SAE2 (91%, 60/66) were detected in most of the tumor fractions (Fig. 4a, b). Expression of SAE2 correlated with EGFR (Pearson *r* = 0.454, 95% confidential interval was between 0.238 and 0.627, and *R*^2^ = 0.206). Pathologically, SAE2 was identified in the tumor nests (302/372, 81.2%) (Fig. 4c1–c4). In NTLT, SAE2 signal was detected in type II pneumocytes (Fig. 4c5). SAE2 was also detected in 87.77% (244/278) of metastatic lymph nodes (data not shown). Statistical analysis showed that SAE2 expression correlated with cigarette smoking, tumor stage, and lymphovascular involvement (Table 1), suggesting that SAE2 expression was closely associated with metastatic potential. Interestingly, among the 302 patients who had high SAE2, 132 (43.7%) had tumor recurrence. Among the 70 patients who had low SAE2, 15 had tumor recurrence (22.8%). All recurrences were detected within 24 months after operation. The recurrence risk of high SAE2 was 2.85-fold higher than that of low SAE2 (*P* < 0.001). Survival rate of low SAE2 patients was better than that of high SAE2 patients (Fig. 4d1). The hazard ratio was 1.708, and the difference in cumulative survival was significant (*P* = 0.002). Multivariate analysis showed that the difference in SAE2 expression was significant between the two groups (*P* = 0.0047). Survival rate of stage I patients with low SAE2 level was also better (Fig. 4d2, *P* = 0.0053). The hazard ratio was 4.08.

**Table 1:** Correlation of SAE2 expression with clinicopathological parameters in patients with LADC

	Expression of SAE2		*P* value	
Parameter	High (*n* = 302)	Low (*n* = 70)	Univariate	Multivariate
Gender				
Male (*n* = 292)	243	49	0.0549^†^	0.107
Female (*n* = 80)	59	21		
Cigarette smoking				
Smoker (*n* = 266)	227	39	0.0012^†^	0.0025
Non-smoker (*n* = 106)	75	31		
Stage				
I (*n* = 83)	65	18	0.042^‡^	0.091
II (*n* = 109)	81	28		
IIIa (*n* = 180)	156	24		
Cell differentiation				
Well (*n* = 56)	42	14	0.205^‡^	0.41
Moderate (*n* = 211)	172	39		
Poor (*n* = 105)	88	17		
Lymphovascular invasion				
Positive (*n* = 278)	244	34	< 0.001^†^	0.0021
Negative (*n* = 94)	58	36		

^†^Two-sided *P* value determined by *χ*^2^ test

^‡^Two-sided *P* value determined by *χ*^2^ test for trend

### Expression of CCDC66 cirRNA in LADC cells

**Fig. 5:**
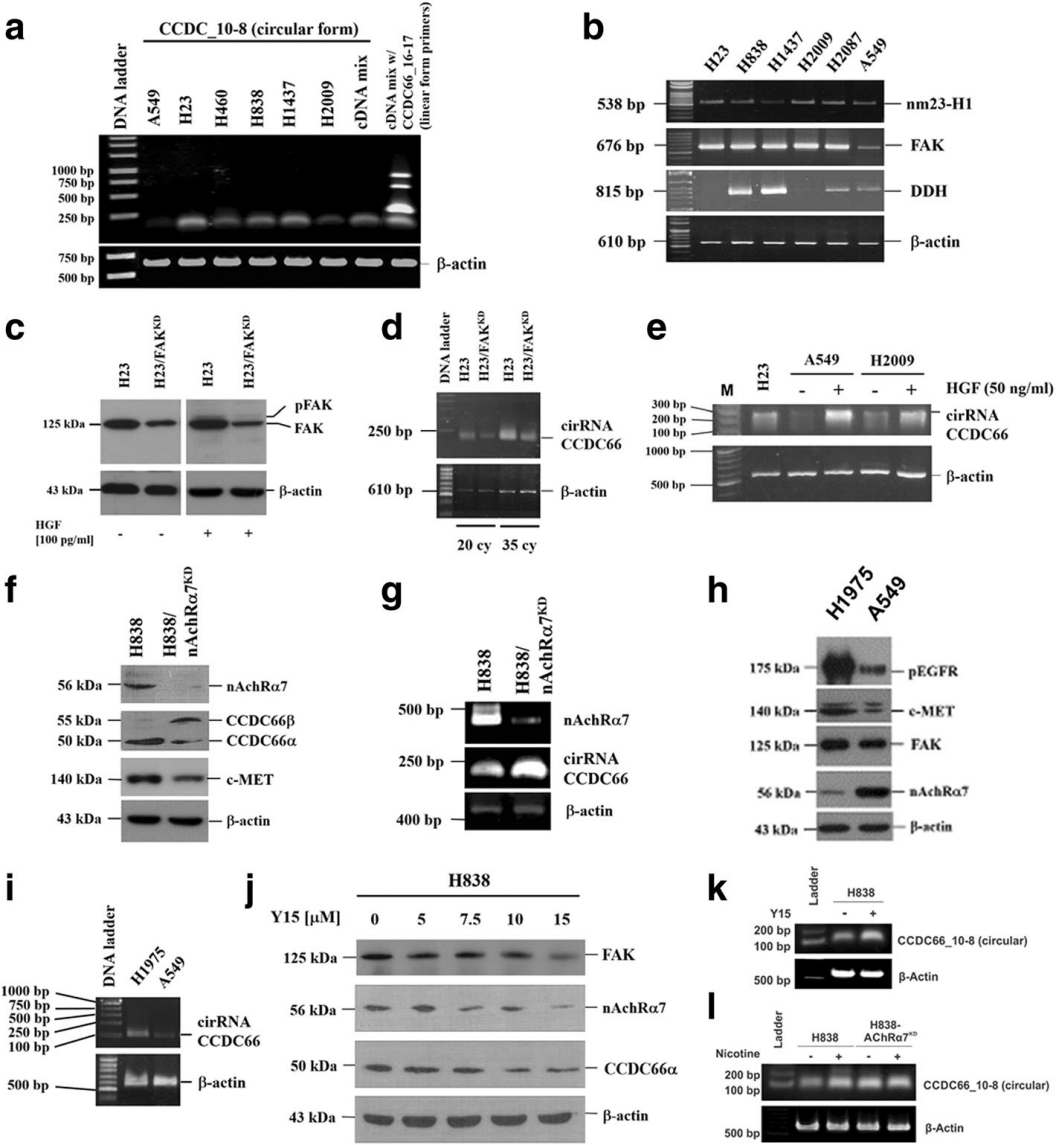
Detection of cirRNA CCDC66 expression in LADC cells. **a** Expression of cirRNA CCDC66 was detected by RT-PCR in LADC cells. **b** Its expression correlated with levels of FAK mRNA, a vital marker closely associated with cancer metastasis and EMT. **c** Silencing of FAK expression reduced **d** cirRNA CCDC66. **e** Addition of HGF (50 ng/ml) increased expression of cirRNA CCDC66. **f** Silencing of nicotinic acetylcholine receptor alpha 7 (nAchRα7), in contrast, increased levels of CCDC66β and **g** cirRNA CCDC66. **h** Comparing gene expression patterns of pEGFR, c-MET, and FAK in EGFR-mutated H1975 and wild-type A549 cells. **i** Expression of pEGFR, c-MET, and FAK was higher in EGFR-mutated H1975 cells. Level of nAchRα7, in contrast, was higher in A549 cells. i Expression of cirRNA CCDC66 was also higher in H1975 cells. **j** Addition of FAK inhibitor Y15 (1,2,4,5-benzenetetramine tetrahydrochloride) inhibited expression of FAK, nAchR7α, and CCDC66α. Y15 inhibited expression of nAchR7α at a concentration of 7.5 μM, CCDC66α at a concentration of 10 μM, and FAK at a concentration of 15 μM. **k** Y15, however, increased cirRNA CCDC66 expression. **l** Addition of nicotine also increased cirRNA CCDC66 expression

As shown in Fig. 3b, c, the increase of protein synthesis before elevation of HIF-1α following exposure to hypoxia suggested that translation efficacy might be increased by reduction of functional microRNA (miRNA), which repressed mRNA translation (a list of potential miRNA is shown in Additional file 1). Recently, Hsiao et al. showed that cirRNA CCDC66 could promote cancer cell growth and metastasis by adsorbing miRNA [24]. Interestingly, cirRNA CCDC66 was also detected in LADC cells (Fig. 5a), and its expression correlated with the levels of FAK mRNA, a vital marker closely associated with cancer metastasis and EMT (Fig. 5b). Silencing of FAK expression (Fig. 5c) reduced cirRNA CCDC66 (Fig. 5d). Addition of HGF, on the contrary, increased expression of cirRNA CCDC66 (Fig. 5e). It is worth noting that silencing of nicotinic acetylcholine receptor alpha 7 (nAchRα7) increased levels of CCDC66β (Fig. 5f) and cirRNA CCDC66 (Fig. 5g), suggesting that nAchRα7 could be a feedback mechanism of HGF-c-MET circuit. Our results showed that expression of pEGFR, c-MET, and FAK were higher in EGFR-mutated H1975 than that in A549 cells (Fig. 5h). Expression of cirRNA CCDC66 was also higher in H1975 cells (Fig. 5i). Levels of nAchRα7, in contrast, were higher in A549 cells. Addition of FAK inhibitor Y15 (1,2,4,5-benzenetetramine tetrahydrochloride, Merck KGaA, Darmstadt, Germany) inhibited expression of FAK, nAchR7α, and CCDC66α (Fig. 5j), however, increased cirRNA CCDC66 expression (Fig. 5k, l).

### Relationship between expression of cirRNA CCDC66 and EMT markers in LADC cells

**Fig. 6:**
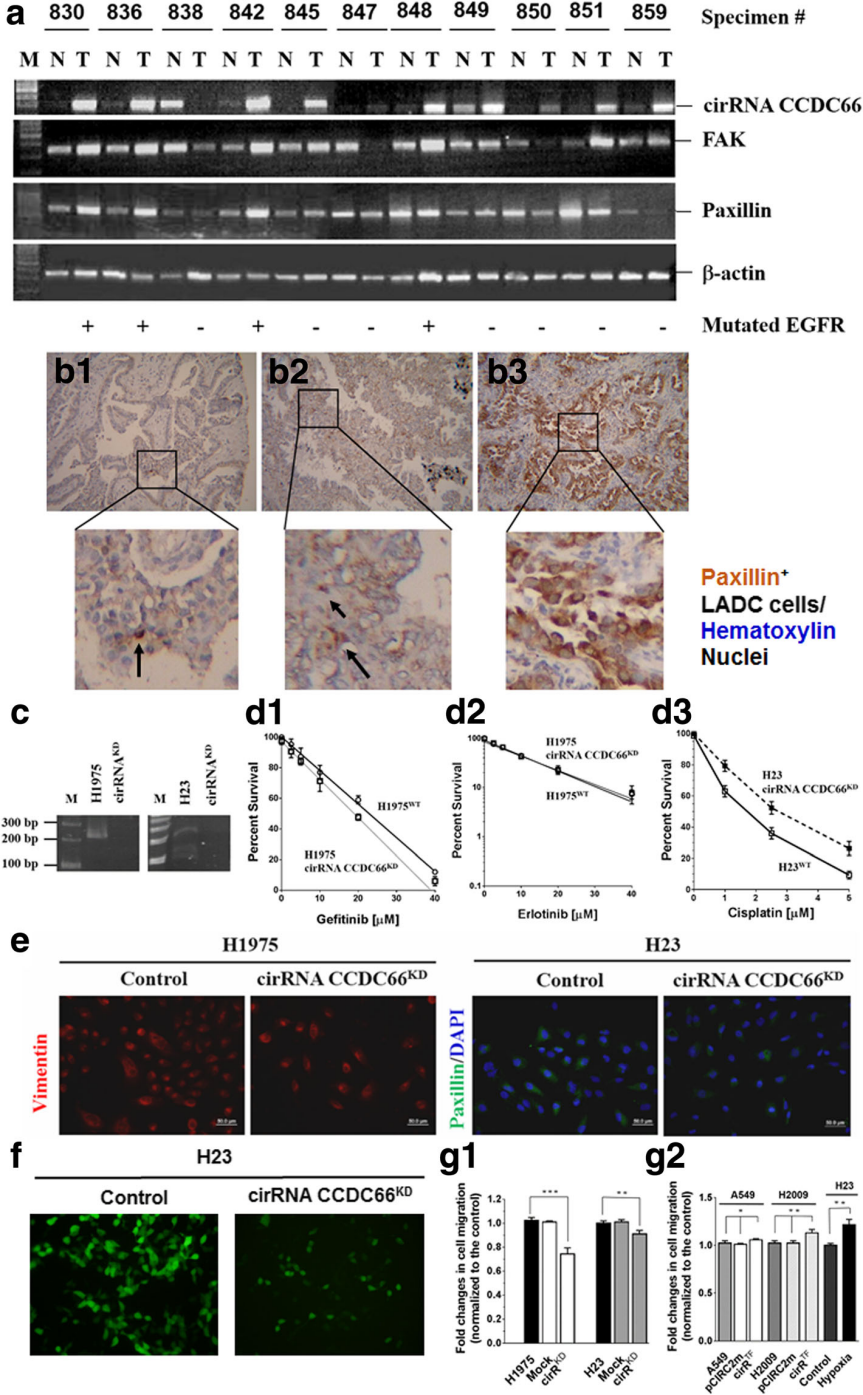
Expression of cirRNA CCDC66 correlated with that of FAK and paxillin, and knockdown of cirRNA CCDC66 reduced expression of EMT markers and invasion ability of LADC cells. **a** Expression of cirRNA CCDC66, FAK, and paxillin was determined by RT-PCR in 11 pairs of LADC specimens. All three tumor makers were detected in most of the tumor fractions [cirRNA CCDC66 (82%, 9/11), FAK (82%, 9/11), and paxillin (91%, 10/11)]. Paxillin was detected in tumor nests (**b1**–**b3**; **b3**, #836) and the neighboring areas (**b2**, #838) by an immunohistochemical staining. Paxillin-positive cells were in dark brown color (indicated by an arrow in b1, #859), showing EMT-like fibroblasts characteristics. **c** Knockdown of cirRNA CCDC66 (cirRNA CCDC66^KD^) expression in H1975 and H23 cells. Resistance to gefitinib (**d1**) or erlotinib (**d2**) was not markedly affected in cirRNA CCDC66^KD^ H1975 cells (**d1, d2**), but clearly increased cisplatin resistance in cirRNA CCDC66^KD^ H23 cells (**d3**). **e** Knockdown of cirRNA CCDC66 expression, however, clearly diminished expression of EMT markers (left panel, H1975 cells were stained for vimentin, red fluorescence; right panel, H23 cells were stained for paxillin, green fluorescence. Nuclei were stained with DAPI, blue fluorescence). **f** Silencing of cirRNA CCDC66 expression markedly reduced cell mobility across extracellular matrix protein-coated carbonate membrane. H23 cells were labeled with green fluorescence protein prior to Matrigel transmembrane assay. Cells in bottom wells of the Boyden chamber were directly detected by green fluorescence. Nuclei were stained with DAPI (blue fluorescence). **g** Effect of cirRNA CCDC66 on cell invasion ability. **g1** Invasion ability of cirRNA CCDC66^KD^, H1975, and H23 cells decreased markedly. Statistical differences were significant (H1975 vs. Mock vs. cirRNA CCDC66^KD^ H1975, *P* < 0.0001 [***], ANOVA; WT H23 vs. Mock vs. cirRNA CCDC66^KD^ H23 cells, *P* = 0.0048 [**], ANOVA). [*P < 0.05; **P < 0.01; ***P < 0.001]; **g2** When cirRNA CCDC66 was overexpressed in A549 cells, the invasion ability increased a little (wild-type vs. pCIRC2-mCherry-transfected [pCIRC2m] vs. pCIRC2-cirRNA CCDC66-transfected A549 cells [cirR^TF^], *P* = 0.037 [*], ANOVA; wild-type vs. cirR^TF^ A549 cells, *P* = 0.1054, *t* test). Comparing wild-type, pCIRC2m, and cirR^TF^ H2009 cells, increment of invasion ability was significant (*P* = 0.0025 [**], ANOVA; H2009 vs. cirR^TF^ H2009, *P* = 0.0137 [*], *t* test). H23 cells exposed to 24-h hypoxia were used as internal comparison parameter (*P* = 0.0031, *t* test)

Expression of cirRNA CCDC66, FAK, and paxillin was determined by RT-PCR in 11 pairs of NTLT and LADC specimens. All three makers were detected in most of the tumor fractions [cirRNA CCDC66 (82%, 9/11), FAK (82%, 9/11), and paxillin (91%, 10/11)] (Fig. 6a). Pathologically, paxillin was identified in tumor nests and the neighboring area (Fig. 6b1–b3), supporting that paxillin expression could correlate with metastatic potential. Knockdown of cirRNA CCDC66 (cirRNA CCDC66^KD^) expression (Fig. 6c) did not affect resistance to gefitinib or erlotinib in H1975 cells (Fig. 6d1, d2) but increased cisplatin resistance in H23 cells (Fig. 6d3). In cirRNA CCDC66^KD^ cells, expression of EMT markers (Fig. 6e) and invasion ability (Fig. 6f, g1) were significantly abrogated. In pCIRC2-cirRNA CCDC66-transfected A549 cells (cirR^TF^), although invasion ability increased slightly (compared to cirR^TF^ H2009 cells), the increment was significant (Fig. 6g2).

## Discussion

EGFRs, especially those with tyrosine kinase mutations, have recently become major targets of TKIs, e.g., gefitinib, erlotinib, and afatinib, in LADC chemotherapy [4, 5, 25]. However, patient’s responses are equivocal [5]. The fact that TKI therapy improves progression-free but not overall survival [25–27] has raised sincere queries into drug’s effectiveness, which may be affected by other factors. Kasahara et al. showed that LADC patients with higher HGF level were more resistant to TKIs [26]. Yano et al. demonstrated that gefitinib resistance was mediated via HGF/c-Met circuit to activate PI3K/AKT [25]. Moreover, continuous exposure of tumor cells to TKIs transpires extra EGFR mutations and drug resistance [27], suggesting nuclear events of DNA sequence alteration and gene activation. Nonetheless, induction mechanisms of gene mutations and phenotype changes, e.g., increase of EMT and metastatic potential [28, 29], are yet to be resolved.

Kessler et al. had found that higher metastatic potential of cancer cells was correlated with expression of SAE1/2 [15]. It was further demonstrated that the elevated metastatic potential was activated by SUMOylation of Rac1, cAMP response element binding (CREB) protein, eEF2, and SIRT1 to increase EMT and cell migration [10, 16–18]. By showing that both EGFR and SAE2 are highly expressed in LADC, and that SAE2 expression correlates with patient’s poor outcomes, our data support their observations that SUMOylation is crucial for tumor progression. In particular, via HGF, hypoxia could enhance SUMOylation of these factors [10, 17, 18]. Among these, SUMOylated Rac1 could further induce expression of a panel of genes, including SNAI (snail family zinc finger) 1, VEGF, and N-cadherin, to facilitate EMT and metastasis of cancer cells [30, 31].

Hypoxia increased expression of EGFR, SAE2, and EMT markers, such as paxillin and vimentin as well [10]. Since silencing of SAE2 (SAE2^KD^) inhibited hypoxic effects, our data suggest that SAE2 is able to maintain protein stability through protein SUMOylation. Moreover, because addition of actinomycin D did not inhibit hypoxic effects, our data also suggest that hypoxia may increase protein synthesis from preexisted mRNA. The rapid rise (0.5 to 1 h, such response was therefore named as immediate reaction) of protein levels before evident accumulation of HIF-1α supported our findings especially that the increased translational efficacy might be initiated before upregulation of transcription (compared to the immediate reaction, such response was named as delayed reaction).

In fact, the different increase rate of respective proteins suggested that the differential augmentation of translation efficacy could be a result of reducing levels of suppressive microRNA (miRNA) following hypoxia (a list of potential miRNA is shown in Additional file 1). However, we did not find an expression surge of specific miRNA under hypoxia. Recently, circular RNA (cirRNA) CCDC66 was shown to promote colon cancer cell growth and metastasis possibly by adsorbing miRNA, which had inhibited expression of several nuclear gene regulators, including DNA methyltransferase 3β (DNMT3B), enhancer of zeste homolog 2 (EZH2), c-Myc, and Yes-associated protein 1 (YAP1) [24]. Showing that cirRNA CCDC66 was expressed in LADC cells, and its expression correlated with FAK, a marker that was associated with cancer metastasis, our results supported their findings. Moreover, silencing of FAK (FAK^KD^) reduced cirRNA CCDC66. Knockdown of nAChRα7 (nAChRα7^KD^), on the other hand, increased cirRNA CCDC66 expression, even in the presence of Y15, which inhibited nAChRα7 expression at low concentrations. The results suggested that the nAChRα7 effect on cirRNA CCDC66 expression was prior to that of FAK.

In addition to cirRNA, miRNA levels are also regulated by the miRNA maturation process. Argonaute 2 (AGO2), a member of RNA-induced silencing complex (RISC), was inhibited by activated EGFR to decelerate miRNA maturation [32]. Moreover, like AGO2, SAE2 and cirRNA CCDC66 were also located on the endoplasmic reticulum (ER) (intracellular localization of SAE2 and cirRNA CCDC66 is shown in Additional files 2, 3, and 4), suggesting that the miRNA maturation and miRNA adsorption process could be carried out at the same time on the ER. Hypoxia-increased buildup of AGO2 was also located on the membrane of intracellular vesicles, which removed miRNA-associated gene suppression. Hypoxia also augmented levels of SAE2 and EMT markers [8, 9, 19]. Inhibition of mitochondrial oxidative phosphorylation increased molecular weight of AGO2 too, which co-precipitated with EGFR, indicating that post-translational modification of EGFR was taken place on intracellular vesicles, and these events could occur without the presence of EGF when cells were under hypoxic conditions [32]. Our results showed that HGF/c-Met circuit was accountable for such modification, and this circuit fortuitously activated EGFR. Because HGF-induced phosphorylation did not occur uniquely on tyrosine residues, phosphorylated serine and threonine residues were less sensitive to TKIs. Moreover, joined effects of c-Met and EGFR on inhibiting cancer cell metastatic ability could be carried out by repressing miR-200 family and methylcytosine dioxygenases (also called ten-eleven translocases, TET) [33]. Two proteins, TET1 (235 kDa) and TET2 (224 kDa), have been identified in TET family. Both TET1 and TET2 proteins possess transmembrane and coiled coil domains (as shown in Additional file 5), which could respectively interact with DRP1 and ATAD3A, the essential components of intracellular transport system, to regulate nuclear entry of these two proteins and other DNA repair-related enzymes [9, 23]. Although the nuclear events of these proteins have yet to be elucidated, it is plausible that TKI inhibits EGFR activity as well as reduces ATAD3A and SAE2 expression to limit nuclear entry of DNA repair-related proteins, e.g., human RAD23 homolog A (hHR23A) and ataxia-telangiectasia-mutated (ATM) kinase, to increase DNA damage and mutation frequency [23, 34]. In an ongoing study, we are focusing on these issues.

## Conclusions

From this study, we identify novel downstream substrates of EGFR and c-Met, SAE2, and cirRNA CCDC66, which are also expressed in LADC. Through SUMOylation, SAE2 could maintain stability of other proteins, including ATAD3A and EMT markers, such as vimentin and paxillin, which are crucial for metastatic potential and drug resistance, and these bioparameters correlate with LADC patient’s prognosis. Therefore, multimodality drugs concurrently aiming at several targets in the circuit of putative feedback mechanism of EGFR and drug resistance-related enzymes would probably provide more benefits for cancer patients.

## Methods

### Tissue specimens and NSCLC lines

Some of the patients in this study were from the cohorts used in the previous studies [8, 10]. Briefly, from January 2007 to December 2012, tissue specimens were collected from 628 patients with newly diagnosed non-small cell lung cancer cell (NSCLC). Samples from all patients, for whom at least one follow-up or death was documented, were pathologically confirmed NSCLC. Of the 628 patients, 372 were diagnosed as having LADC. The stage of the disease was classified according to the new international staging system for lung cancer [35]. The Medical Ethics Committee had approved the protocol, and written informed consent was obtained from every patient before surgery (DMR99-IRB-203). All patients had undergone surgical resection and radical N2 lymph node dissection. Tumor size, numbers of lymph node involvement, differentiation, vascular invasion, and mitotic number were evaluated. Patients with lymph node involvement or loco-regional recurrence received irradiation at the afflicted areas. Those with distant metastasis were treated with chemotherapy. After treatment, patients were routinely followed every 3 to 6 months in the outpatient department. Results from blood examination, biochemical studies, chest radiography, abdominal sonography, whole body bone scan, and computerized tomography scans of chest that indicated any evidence of disease were interpreted as tumor recurrence or metastasis. The average age of the male patients (*n* = 205) was 62.8 ± 12.49 years and that of the female patients (*n* = 167) was 58.9 ± 9.41 years (*t* test, *P* < 0.001). Immunohistochemical staining was carried out using a single-blind procedure.

Eight NSCLC cell lines (H125, H23, H226, H838, H1437, H2009, H2087, and A549) from American Type Culture Collection (ATCC, Manassas, VA**)** were used for evaluation of gene expression. H125, H23, H838, H1437, H2009, H2087, and A549 are LADC cells, and H226 is an epithelial cell type. Cells were grown at 37 °C in a monolayer in RPMI 1640 supplemented with 10% fetal calf serum (FCS), 100 IU/mL penicillin, and 100 μg/mL streptomycin.

### RT-PCR

Reverse transcription-polymerase chain reaction (RT-PCR) was performed as described before [6–10]. Following extraction of total RNA and synthesis of the first-strand cDNA, an aliquot of cDNA was subjected to 35 cycles of PCR to determine the integrity of β-actin mRNA [7]. The cDNA used in RT-PCR was adjusted according to the quality and quantity of β-actin mRNA.

The primer sequences were selected by Primer3 (http://bioinfo.ut.ee/primer3/).

For FAK, the primers are FAKs: 5′-TGAATTTCTTCTATCAACAG-3′ [sense primer, nts 485-504, NM_001352746] and FAKa: 5′-CCTGGCTTCATCTATTCCAT-3′ [antisense primer, nts 1142-1161]. The anticipated DNA fragment is 677 base-pair (bp). For SAE2, the primers are SAE2s: 5′-TGAAAGTGGAACAGCTGGGT-3′ [nts 481-500, NM_005499] and SAE2a: 5′-GCAGGAATAATGTTCCCTGCC-3′ [nts 1207-1227]. The anticipated cDNA fragment is 847 bp. For nAchRα7, the primers are nAchRα7s: 5′-GCCGCAGGACGCTCTACTAT-3′ [nts 912-931, NM_148911] and nAchRα7a: 5′-ACGGCACTCATCTCCACACT-3′ [nts 1334-1353]. The anticipated DNA fragment is 442 base-pair (bp). For cirRNA CCDC66 (circular form_10-8, exons 10 to 8), the primers are CCDC66s: 5′-TCTCTTGGACCCAGCTCAG-3′ [nts 1428-1446, NM_001141947] and CCDC66a: 5′-TGAATCAAAGTGCATTGCCC-3′ [nts 1175-1194]. The anticipated DNA fragment is 272 bp. For CCDC66 mRNA (linear form_16-17, exons 16 to 17), the primers are CCDC66s: 5′-ACCTTGATCCCGATGCACC-3′ [nts 2657-2675, NM_001141947] and CCDC66a: 5′-ACGGCACTCATCTCCACACT-3′ [nts 2801-2820]. The anticipated DNA fragment is 164 bp. For β-actin, the primers are β-actins: 5′-AGA GCT ACG AGC TGC CTG AC-3′ [nts 797-816, NM_001101] and β-actins: 5′-CAC CTT CAC CGT TCC AGT TT-3′ [nts 1337-1356]. The anticipated DNA fragment is 560 bp.

### Immunoblotting analysis and immunocytochemistry

Immunoblotting and immunohistochemistry were performed as described previously [6]. Antibodies for β-actin were obtained from Chemicon International (Temecula, CA). Antibodies to EGFR, ATAD3A, DRP1, and SAE2 were home-made and characterized in the lab (for SAE2 antibody characterization, please refer to Additional files 6 and 7). For immunocytochemistry, the cells were grown overnight on slides and fixed with cold methanol/acetone for 10 min before staining. Immunological staining was performed by an immunoperoxidase method [6–10]. SAE2 antibodies were not added in the negative control group.

### Silencing of gene expression using lentivirus-carrying shRNA

Lentivirus carrying shRNA sequence to the target gene was prepared using a three-plasmid transfection method [36] and used to infect cells. Cells with gene knockdown (SAE2^KD^, EGFR^KD^, FAK^KD^, c-Met^KD^ or nAchRα7^KD^) were selected using 1 μg/mL puromycin. The siRNA used for silencing gene expression was obtained from the National RNAi Core Facility (Institute of Molecular Biology/Genomic Research Centre, Academia Sinica, Taipei, Taiwan). Knockdown of cirRNA CCDC66 (cirRNA CCDC66^KD^) was carried out by direct delivery of siRNAs (siJCT1 and siJCT2) [24] into H23 and H1975 cells using Ambion Silencer® siRNA transfection kit [10]. Reduction of cirRNA CCDC66 was determined by RT-PCR and gel electrophoresis.

### Drug sensitivity assay

Drug sensitivity was measured by a WST-1 assay [37]. Cells were seeded at different cell numbers in a 96-well plate 18 h prior to drug challenge. Cells were pulse-treated with various concentrations of cisplatin for 4 h. The control cells were treated with the solvent of the drug. Total survival of the cells was determined 72 h following drug challenge, and percent survival was estimated by dividing optical absorbance resulted from each experiment group with that of the control group. Each experiment was done in triplicates, and the optical absorbance was measured by coloration of reacted substrate, WST-1 (BioVision, Mountain View, CA), which was catalyzed by mitochondrial dehydrogenases.

### Matrigel invasion assay

Matrigel invasion assay was performed following the protocol suggested by BD Biosciences (Bedford, MA, USA). Briefly, a vial of BD Matrigel™ basement membrane matrix (BD-MBM, 356234) was thawed on ice overnight and diluted to ½ and ¼ with ice-cold serum-free Dulbecco’s modified Eagle’s medium (DMEM). Five milliliters of the diluted BD-MBM was spread to a petri dish on ice, before a piece of polycarbonate membrane (with 5.0-μm pore size) was submerged into the suspension mixture. Membrane coating was carried out at room temperature for 1 h. The membrane was rinsed with serum-free DMEM once and mounted onto the Boyden chamber. The lower chamber contained the full medium. 1 × 10^5^ cells were pipetted into the well of the upper chamber at a 1-h interval for 8 h and incubated at 37 °C for 24 h in a humidified incubator with 5% CO_2_. Following complete removal of non-invading cells, the membrane was lifted from the chamber and fixed in 100% methanol for 2 min. Cells on the membrane were stained with 1% toluidine blue for 2 min and washed twice with distilled water. After counting cells, percent invasion on membrane were calculated by comparing the experimental to the control group. Some assays were done by green fluorescence-labeled cells.

### Slide evaluation of SAE2 expression by immunohistochemical staining

In each pathological section, non-tumor lung tissue (NTLT) served as the internal negative control. Slides were evaluated by two independent pathologists blinded to the clinicopathological knowledge. The Immunoreactive Scoring System was adapted for this study [38]. A specimen was considered having strong signals when more than 50% of cancer cells were positively stained; intermediate, if 25–50% of the cells stained positive; weak, if less than 25% or more than 10% of the cells were positively stained; and negative, if less than 10% of the cancer cells were stained. Cases with strong and intermediate signals were classified as SAE2^+^. Those with weak or negative signals were classified as SAE2^−^ [6–10].

### Statistical analysis

Correlation between SAE2 expression and clinicopathological factors was analyzed by either a chi-square test or a chi-square test for trend. Survival curves were plotted using the Kaplan-Meier estimator [39] and analyzed by the log-rank test [40]. Statistical analysis was performed using GraphPad Prism6 (La Jolla, CA). Statistical significance was set at *P* < 0.05.

## Additional files

Additional file 1: The potential microRNA (miRNA) which could suppress protein synthesis of paxillin, vimentin, HIF-1α, SAE2, and EGFR (the data was obtained from the published data or a web search by using online software programs, miRanda [http://34.236.212.39/microrna/home.do]). (DOCX 26 kb)

Additional file 2: Prediction of transmembrane domain in amino acid sequences of SAE2. (DOCX 25 kb)

Additional file 3: The intracellular location of SAE2 and eEF2 in A549 LADC cells as determined by fluorescence immunocytochemical staining. (DOCX 294 kb)

Additional file 4: The intracellular location of cirRNA CCDC66 in A549 LADC cells as determined by fluorescence immunocytochemical staining. (DOCX 523 kb)

Additional file 5: Prediction of transmembrane and coiled-coil domains in amino acid sequences of TET1 and TET2. (DOCX 335 kb)

Additional file 6: Characterization of monoclonal antibodies to SAE2 and the immunoprecipitated products. (DOCX 807 kb)

Additional file 7: Supplemental methods. (DOCX 20 kb)

## Abbreviations

AIF: Apoptosis-inducing factor
ATAD3A: ATPase family, AAA domain-containing 3A
ATM: Ataxia-telangiectasia-mutated kinase
CCDC66: Coiled-coil domain containing 66
CIP: Calf intestinal phosphatase
cirRNA: Circular RNA
EGFR: Human epidermal growth factor receptor (HER-1, or ErbB-1)
EMT: Epithelial-to-mesenchymal transition
HGF: Hepatocyte growth factor
LADC: Lung adenocarcinoma
MALDI-TOF: Matrix-assisted laser desorption/ionization time of flight mass spectrometry
NSCLC: Non-small cell lung carcinoma
pCIRC2-cirRNA CCDC66: Plasmid containing cDNA insert for expressing cirRNA CCDC66
PI3K: Phosphatidylinositol 3-kinase
RT-PCR: Reverse transcription-polymerase chain reaction
SAE1: SUMO-activating enzyme subunit 1
SAE2: SUMO-activating enzyme subunit 2
SNAI 1: Snail family zinc finger 1
SUMO: Small ubiquitin-related modifier
TET: Ten-eleven translocases
TKIs: Tyrosine kinase inhibitors
