# Supplementary material for: The role of HGF-MET pathway and CCDC66 cirRNA expression in EGFR resistance and epithelial-to-mesenchymal transition of lung adenocarcinoma cells
Source: J Hematol Oncol. 2018 May 31;11:74. doi: 10.1186/s13045-018-0557-9 (PMC5984410; doi:10.1186/s13045-018-0557-9)
Supplement: Supplementary file 1 — The potential microRNA (miRNA) which could suppress protein synthesis of paxillin, vimentin, HIF-1α, SAE2, and EGFR (the data was obtained from the published data or a web search by using online software programs, miRanda [http://34.236.212.39/microrna/home.do]). (DOCX 26 kb) [file 13045_2018_557_MOESM1_ESM.docx]

**Additional file 1** The potential microRNA (miRNA) which could suppress protein synthesis of paxillin, vimentin, HIF-1α, SAE2 and EGFR was obtained from the published data or a web search by using online software programs, miRanda (www.microrna.org).

| **Genes** | published miRNA | **Reference** |
| --- | --- | --- |
| EGFR | miR-320b | Wang *et al.*^1^ |
| paxillin | miR-218 | Wu *et al.* ^2^ |
| p-c-Met | miR-21  miR-34  miR-199a*  miR-424 | Hwang *et al.*^3^  Tanaka *et al.*^4^  Kim *et al.*^5^  Das *et al.*^6^ |
| SAE2 | [hsa-miR-527](http://www.genecards.org/cgi-bin/urlredir.pl?gene=UBA2&company=sabio&type=mir&url=https%3A%2F%2Fwww%2Eqiagen%2Ecom%2Fgeneglobe%2Fmiprimerview%2Easpx%3FID%3DMS00033866) (miRanda) | http://mirnamap.mbc.nctu.edu.tw/ |
| SOX2 | miR-126  miR-145 | Otsubo *et al*.^7^  Xu *et al*.^8^ |
| Vimentin | miR-21  miR-30a | Cheng *et al.*^9^  Yamada *et al.*^10^ |

**References**

1. [Wang L](http://www.ncbi.nlm.nih.gov/pubmed?term=Wang%20L%5BAuthor%5D&cauthor=true&cauthor_uid=24083596), [Yao J](http://www.ncbi.nlm.nih.gov/pubmed?term=Yao%20J%5BAuthor%5D&cauthor=true&cauthor_uid=24083596), [Shi X](http://www.ncbi.nlm.nih.gov/pubmed?term=Shi%20X%5BAuthor%5D&cauthor=true&cauthor_uid=24083596), [Hu L](http://www.ncbi.nlm.nih.gov/pubmed?term=Hu%20L%5BAuthor%5D&cauthor=true&cauthor_uid=24083596), [Li Z](http://www.ncbi.nlm.nih.gov/pubmed?term=Li%20Z%5BAuthor%5D&cauthor=true&cauthor_uid=24083596), [Song T](http://www.ncbi.nlm.nih.gov/pubmed?term=Song%20T%5BAuthor%5D&cauthor=true&cauthor_uid=24083596), et al. MicroRNA-302b suppresses cell proliferation by targeting EGFR in human hepatocellularcarcinoma SMMC-7721 cells. [BMC Cancer](http://www.ncbi.nlm.nih.gov/pubmed/?term=MicroRNA-302b+suppresses+cell+proliferation+by+targeting+EGFR+in+human+hepatocellular+carcinoma+SMMC-7721+cells). 2013;13:448.
2. [Wu DW](http://www.ncbi.nlm.nih.gov/pubmed?term=Wu%20DW%5BAuthor%5D&cauthor=true&cauthor_uid=21159652), [Cheng YW](http://www.ncbi.nlm.nih.gov/pubmed?term=Cheng%20YW%5BAuthor%5D&cauthor=true&cauthor_uid=21159652), [Wang J](http://www.ncbi.nlm.nih.gov/pubmed?term=Wang%20J%5BAuthor%5D&cauthor=true&cauthor_uid=21159652), [Chen CY](http://www.ncbi.nlm.nih.gov/pubmed?term=Chen%20CY%5BAuthor%5D&cauthor=true&cauthor_uid=21159652), [Lee H](http://www.ncbi.nlm.nih.gov/pubmed?term=Lee%20H%5BAuthor%5D&cauthor=true&cauthor_uid=21159652). Paxillin predicts survival and relapse in non-small cell lung cancer by microRNA-218 targeting. [Cancer Res](http://www.ncbi.nlm.nih.gov/pubmed/21159652). 2010;70:10392-10401.
3. [Hwang JH](http://www.ncbi.nlm.nih.gov/pubmed?term=Hwang%20JH%5BAuthor%5D&cauthor=true&cauthor_uid=20498843), [Voortman J](http://www.ncbi.nlm.nih.gov/pubmed?term=Voortman%20J%5BAuthor%5D&cauthor=true&cauthor_uid=20498843), [Giovannetti E](http://www.ncbi.nlm.nih.gov/pubmed?term=Giovannetti%20E%5BAuthor%5D&cauthor=true&cauthor_uid=20498843), Steinberg SM, Leon LG, Kim YT, et al. Identification of microRNA-21 as a biomarker for chemoresistance and clinical outcome following adjuvant therapy in resectable pancreatic cancer. [PLoS One](http://www.ncbi.nlm.nih.gov/pubmed/?term=Identification+of+MicroRNA-21+as+a+Biomarker+for+Chemoresistance+and+Clinical+Outcome+Following+Adjuvant+Therapy+in+Resectable+Pancreatic+Cancer). 2010;5:e10630.
4. [Tanaka N](http://www.ncbi.nlm.nih.gov/pubmed?term=Tanaka%20N%5BAuthor%5D&cauthor=true&cauthor_uid=23525472), [Toyooka S](http://www.ncbi.nlm.nih.gov/pubmed?term=Toyooka%20S%5BAuthor%5D&cauthor=true&cauthor_uid=23525472), [Soh J](http://www.ncbi.nlm.nih.gov/pubmed?term=Soh%20J%5BAuthor%5D&cauthor=true&cauthor_uid=23525472), [Tsukuda K](http://www.ncbi.nlm.nih.gov/pubmed/?term=Tsukuda%20K%5BAuthor%5D&cauthor=true&cauthor_uid=23525472), [Shien K](http://www.ncbi.nlm.nih.gov/pubmed/?term=Shien%20K%5BAuthor%5D&cauthor=true&cauthor_uid=23525472), [Furukawa M](http://www.ncbi.nlm.nih.gov/pubmed/?term=Furukawa%20M%5BAuthor%5D&cauthor=true&cauthor_uid=23525472), et al. Downregulation of microRNA-34 induces cell proliferation and invasion of human mesothelial cells. [Oncol Rep](http://www.ncbi.nlm.nih.gov/pubmed/?term=Down-regulation+of+microRNA34+induces+cell+proliferation+and+invasion+of+human+mesothelial+cells). 2013;29:2169-2174.
5. [Kim S](http://www.ncbi.nlm.nih.gov/pubmed?term=Kim%20S%5BAuthor%5D&cauthor=true&cauthor_uid=18456660), [Lee UJ](http://www.ncbi.nlm.nih.gov/pubmed?term=Lee%20UJ%5BAuthor%5D&cauthor=true&cauthor_uid=18456660), [Kim MN](http://www.ncbi.nlm.nih.gov/pubmed?term=Kim%20MN%5BAuthor%5D&cauthor=true&cauthor_uid=18456660), [Lee EJ](http://www.ncbi.nlm.nih.gov/pubmed/?term=Lee%20EJ%5BAuthor%5D&cauthor=true&cauthor_uid=18456660), [Kim JY](http://www.ncbi.nlm.nih.gov/pubmed/?term=Kim%20JY%5BAuthor%5D&cauthor=true&cauthor_uid=18456660), [Lee MY](http://www.ncbi.nlm.nih.gov/pubmed/?term=Lee%20MY%5BAuthor%5D&cauthor=true&cauthor_uid=18456660), et al. MicroRNA miR-199a* regulates the MET proto-oncogene and the downstream extracellular signal-regulated kinase 2 (ERK 2). [J Biol Chem. 2008;283:18158-18166.](http://www.ncbi.nlm.nih.gov/pubmed/?term=MicroRNA+miR-199a*+Regulates+the+MET+Proto-oncogene+and+the+Downstream+Extracellular+Signal-regulated)
6. [Das R](http://www.ncbi.nlm.nih.gov/pubmed?term=Das%20R%5BAuthor%5D&cauthor=true&cauthor_uid=25262538), [Gregory PA](http://www.ncbi.nlm.nih.gov/pubmed?term=Gregory%20PA%5BAuthor%5D&cauthor=true&cauthor_uid=25262538), [Hollier BG](http://www.ncbi.nlm.nih.gov/pubmed?term=Hollier%20BG%5BAuthor%5D&cauthor=true&cauthor_uid=25262538), [Tilley WD](http://www.ncbi.nlm.nih.gov/pubmed?term=Tilley%20WD%5BAuthor%5D&cauthor=true&cauthor_uid=25262538), [Selth LA](http://www.ncbi.nlm.nih.gov/pubmed?term=Selth%20LA%5BAuthor%5D&cauthor=true&cauthor_uid=25262538). Epithelial plasticity in prostate cancer: principles and clinical perspectives. [Trends Mol Med](http://www.ncbi.nlm.nih.gov/pubmed/?term=Epithelial+plasticity+in+prostate+cancer%3A+principles+and+clinical+perspectives). 2014;20:643-651.
7. [Otsubo T](http://www.ncbi.nlm.nih.gov/pubmed?term=Otsubo%20T%5BAuthor%5D&cauthor=true&cauthor_uid=21304604), [Akiyama Y](http://www.ncbi.nlm.nih.gov/pubmed?term=Akiyama%20Y%5BAuthor%5D&cauthor=true&cauthor_uid=21304604), [Hashimoto Y](http://www.ncbi.nlm.nih.gov/pubmed?term=Hashimoto%20Y%5BAuthor%5D&cauthor=true&cauthor_uid=21304604), [Shimada S](http://www.ncbi.nlm.nih.gov/pubmed?term=Shimada%20S%5BAuthor%5D&cauthor=true&cauthor_uid=21304604), [Goto K](http://www.ncbi.nlm.nih.gov/pubmed?term=Goto%20K%5BAuthor%5D&cauthor=true&cauthor_uid=21304604), [Yuasa Y](http://www.ncbi.nlm.nih.gov/pubmed?term=Yuasa%20Y%5BAuthor%5D&cauthor=true&cauthor_uid=21304604). MicroRNA-126 inhibits SOX2 expression and contributes to gastric carcinogenesis. [PLoS One](http://www.ncbi.nlm.nih.gov/pubmed/?term=MicroRNA-126+Inhibits+SOX2+Expression+and+Contributes+to+Gastric+Carcinogenesis). 2011;6:e16617.
8. [Xu N](http://www.ncbi.nlm.nih.gov/pubmed?term=Xu%20N%5BAuthor%5D&cauthor=true&cauthor_uid=19409607), [Papagiannakopoulos T](http://www.ncbi.nlm.nih.gov/pubmed?term=Papagiannakopoulos%20T%5BAuthor%5D&cauthor=true&cauthor_uid=19409607), [Pan G](http://www.ncbi.nlm.nih.gov/pubmed?term=Pan%20G%5BAuthor%5D&cauthor=true&cauthor_uid=19409607), [Thomson JA](http://www.ncbi.nlm.nih.gov/pubmed?term=Thomson%20JA%5BAuthor%5D&cauthor=true&cauthor_uid=19409607), [Kosik KS](http://www.ncbi.nlm.nih.gov/pubmed?term=Kosik%20KS%5BAuthor%5D&cauthor=true&cauthor_uid=19409607). MicroRNA-145 regulates OCT4, SOX2, and KLF4 and represses pluripotency in humanembryonic stem cells. Cell. 2009;137:647-658.
9. [Cheng CW](http://www.ncbi.nlm.nih.gov/pubmed?term=Cheng%20CW%5BAuthor%5D&cauthor=true&cauthor_uid=22476851), [Wang HW](http://www.ncbi.nlm.nih.gov/pubmed?term=Wang%20HW%5BAuthor%5D&cauthor=true&cauthor_uid=22476851), [Chang CW](http://www.ncbi.nlm.nih.gov/pubmed?term=Chang%20CW%5BAuthor%5D&cauthor=true&cauthor_uid=22476851), Chu HW, Chen CY, Yu JC, et al. MicroRNA-30a inhibits cell migration and invasion by downregulating vimentin expression and is a potential prognostic marker in breast cancer. [Breast Cancer Res Treat](http://www.ncbi.nlm.nih.gov/pubmed/22476851). 2012;134:1081-1093.
10. [Yamada M](http://www.ncbi.nlm.nih.gov/pubmed?term=Yamada%20M%5BAuthor%5D&cauthor=true&cauthor_uid=24063588), [Kubo H](http://www.ncbi.nlm.nih.gov/pubmed?term=Kubo%20H%5BAuthor%5D&cauthor=true&cauthor_uid=24063588), [Ota C](http://www.ncbi.nlm.nih.gov/pubmed?term=Ota%20C%5BAuthor%5D&cauthor=true&cauthor_uid=24063588), [Takahashi T](http://www.ncbi.nlm.nih.gov/pubmed/?term=Takahashi%20T%5BAuthor%5D&cauthor=true&cauthor_uid=24063588), [Tando Y](http://www.ncbi.nlm.nih.gov/pubmed/?term=Tando%20Y%5BAuthor%5D&cauthor=true&cauthor_uid=24063588), [Suzuki T](http://www.ncbi.nlm.nih.gov/pubmed/?term=Suzuki%20T%5BAuthor%5D&cauthor=true&cauthor_uid=24063588), et al. The increase of microRNA-21 during lung fibrosis and its contribution to epithelial-mesenchymal transition in pulmonary epithelial cells. [Respir Res. 2013;14:95.](http://www.ncbi.nlm.nih.gov/pubmed/?term=The+increase+of+microRNA-21+during+lung+fibrosis+and+its+contribution+to+epithelial-mesenchymal+transition+in+pulmonary+epithelial+cells)
