# Supplementary material for: The role of HGF-MET pathway and CCDC66 cirRNA expression in EGFR resistance and epithelial-to-mesenchymal transition of lung adenocarcinoma cells
Source: J Hematol Oncol. 2018 May 31;11:74. doi: 10.1186/s13045-018-0557-9 (PMC5984410; doi:10.1186/s13045-018-0557-9)
Supplement: Supplementary file 2 — Prediction of transmembrane domain in amino acid sequences of SAE2. (DOCX 25 kb) [file 13045_2018_557_MOESM2_ESM.docx]

**Additional file 2** Prediction of transmembrane domain in amino acid sequences of SAE2.

## ProScale analysis of SAE2 to predict the probable presence of transmembrane domain (TMD) (https://web.expasy.org/protscale/)

## [
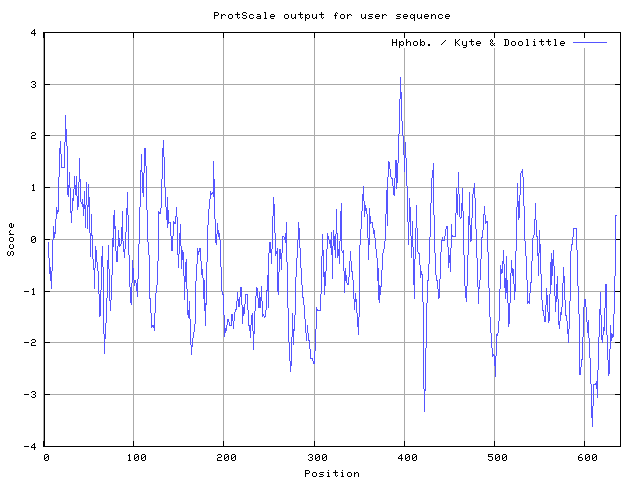
](http://web.expasy.org/tmp/pscale19196.gif)

## B. Analysis of SAE2 by TMHMM to predict the perspective TDM (http://www.cbs.dtu.dk/services/TMHMM-2.0/).


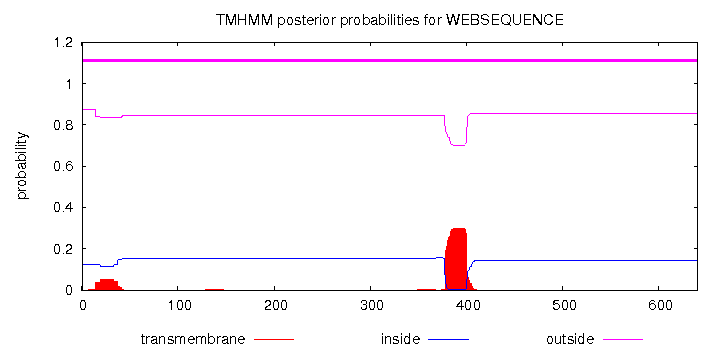


**C. Prediction of transmembrane domain in SAE2 by PSORT II** (http://psort.hgc.jp/form2.html)

[ALOM: Klein et al's method for TM region allocation](http://psort.hgc.jp/psort/helpwww2.html#ealom)

Init position for calculation: 1

Tentative number of TMS(s) for the threshold 0.5: 2

Number of TMS(s) for threshold 0.5: 1

INTEGRAL Likelihood = -4.30 Transmembrane 383 - 399

PERIPHERAL Likelihood = 3.98 (at 36)

ALOM score: -4.30 (number of TMSs: 1)

[MTOP: Prediction of membrane topology (Hartmann et al.)](http://psort.hgc.jp/psort/helpwww2.html#mtop)

Center position for calculation: 390

Charge difference: -1.5 C( 1.0) - N( 2.5)

N >= C: N-terminal side will be inside

**>>> membrane topology: type 2 (cytoplasmic tail 1 to 378; TMD, 379-400)**

370 380 390 400 410 420
LRMHIFSMNM KSRFDIK**SMA** **GNIIPAIATT** **NAVIAGLIVL** EGLKILSGKI DQCRTIFLNK
 transmembrane domain (SAE2, homo sapiens)

850 860 870 880 890 900
PADKHKTKGI **AGKIIPALVT** **TTAVVAGFVC IELI**KVIQNK ALEKYKSTFM NLGIPFFGFV
 transmembrane domain (UBA1, Dictyostelium discoideum)

## Additional file 2 Prediction of transmembrane domain in amino acid sequences of SAE2.

## A: Using ProScale (http://web.expasy.org/protscale/) to predict the transmembrane domain (hydrophobicity of a stretch of amino acids is greater than 3) in amino acid sequences of SAE2. B: Using a web program (Prediction of transmembrane helices in proteins,

http://www.cbs.dtu.dk/services/TMHMM-2.0/) to predict the transmembrane domain in amino acid sequences of SAE2.

**C**: Using PSORT II (<http://psort.hgc.jp/form2.html>) to predict the transmembrane domain in amino acid sequences of SAE2.
