# Supplementary material for: The role of HGF-MET pathway and CCDC66 cirRNA expression in EGFR resistance and epithelial-to-mesenchymal transition of lung adenocarcinoma cells
Source: J Hematol Oncol. 2018 May 31;11:74. doi: 10.1186/s13045-018-0557-9 (PMC5984410; doi:10.1186/s13045-018-0557-9)
Supplement: Supplementary file 3 — The intracellular location of SAE2 and eEF2 in A549 LADC cells as determined by fluorescence immunocytochemical staining. (DOCX 294 kb) [file 13045_2018_557_MOESM3_ESM.docx]

**Additional file 3** The intracellular location of SAE2 and eEF2 in A549 LADC cells as determined by fluorescence immunocytochemical staining.

**
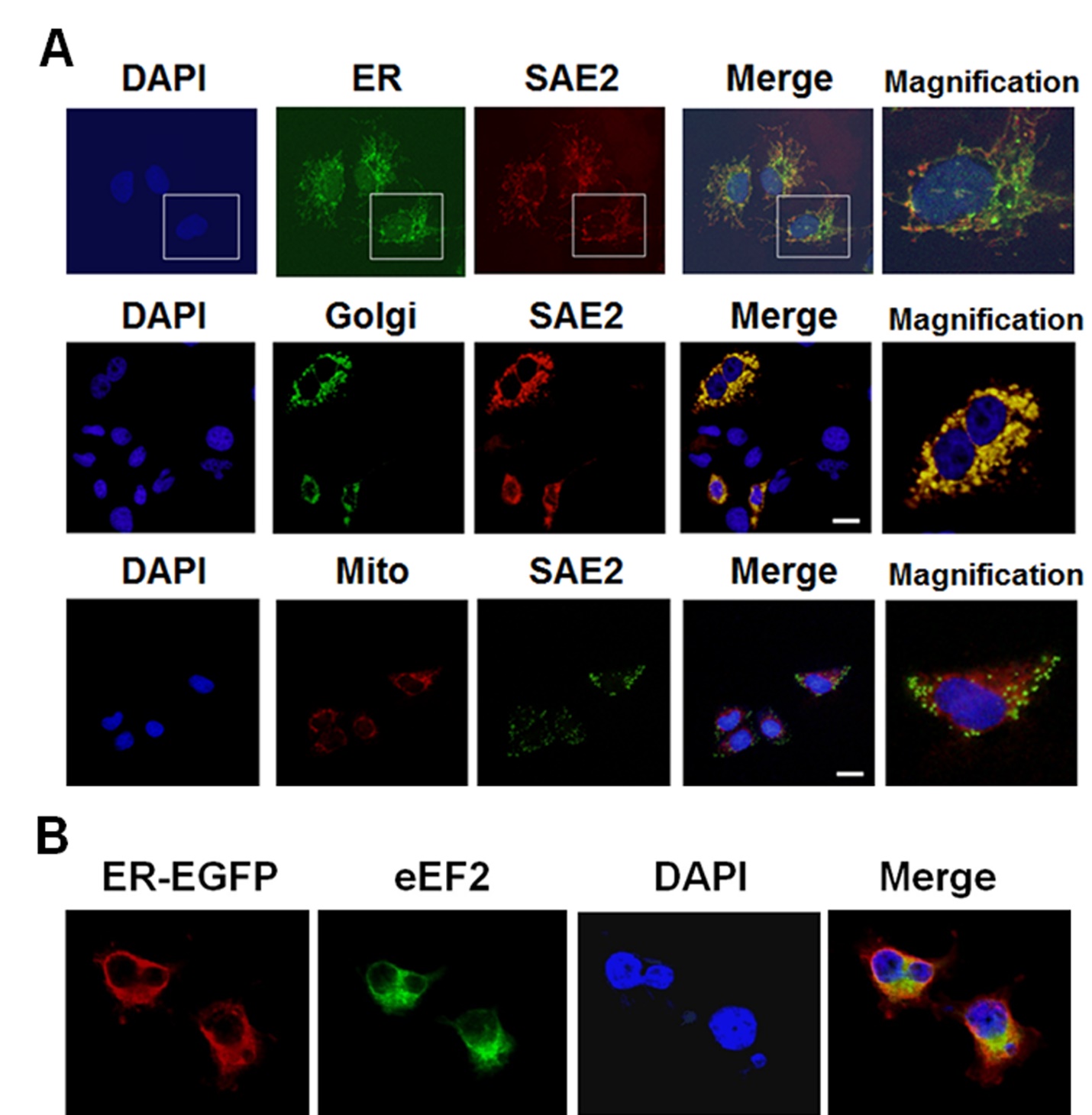
**

**Additional file 3** Fluorescence immunocytochemical staining of A549 LADC cells. **A** SAE2 was detected by specific monoclonal antibodies, and visualized by the secondary antibodies conjugated with Texas red (the first and the second rows) or FITC (the third row). The endoplasmic reticulum (ER) was labelled using plasmids encoding ER-targeted Discosoma green fluorescent protein (ER-GFP), and the Golgi apparatus was labelled by transfecting A549 cells with plasmids containing galactosyltransferase-GFP (GT-GFP). Nuclei were stained with fluorescent dye 4', 6-diamidino-2-phenylindole (DAPI, blue fluorescence), and the mitochondria were labelled with MitoTracker^®^ Red CMXRos dye. A merged image of the first, second and third column is shown in the fourth column, and the magnification of a specific cell confirms that SAE2 is mainly located in the ER and the Golgi apparatus. The white bar represents 20 μm. **B** Intracellular eEF2 was identified by specific monoclonal antibodies, and visualized by the secondary antibodies conjugated with FITC (green fluorescence). The ER was labelled by transfecting A549 cells with plasmids encoding ER-targeted Discosoma enhanced green fluorescent protein (ER-EGFP, red fluorescence), showing that eEF2 was co-localized with the ER.
