# Supplementary material for: The role of HGF-MET pathway and CCDC66 cirRNA expression in EGFR resistance and epithelial-to-mesenchymal transition of lung adenocarcinoma cells
Source: J Hematol Oncol. 2018 May 31;11:74. doi: 10.1186/s13045-018-0557-9 (PMC5984410; doi:10.1186/s13045-018-0557-9)
Supplement: Supplementary file 4 — The intracellular location of cirRNA CCDC66 in A549 LADC cells as determined by fluorescence immunocytochemical staining. (DOCX 523 kb) [file 13045_2018_557_MOESM4_ESM.docx]

**Additional file 4** The intracellular location of cirRNA CCDC66 in A549 LADC cells as determined by fluorescence immunocytochemical staining


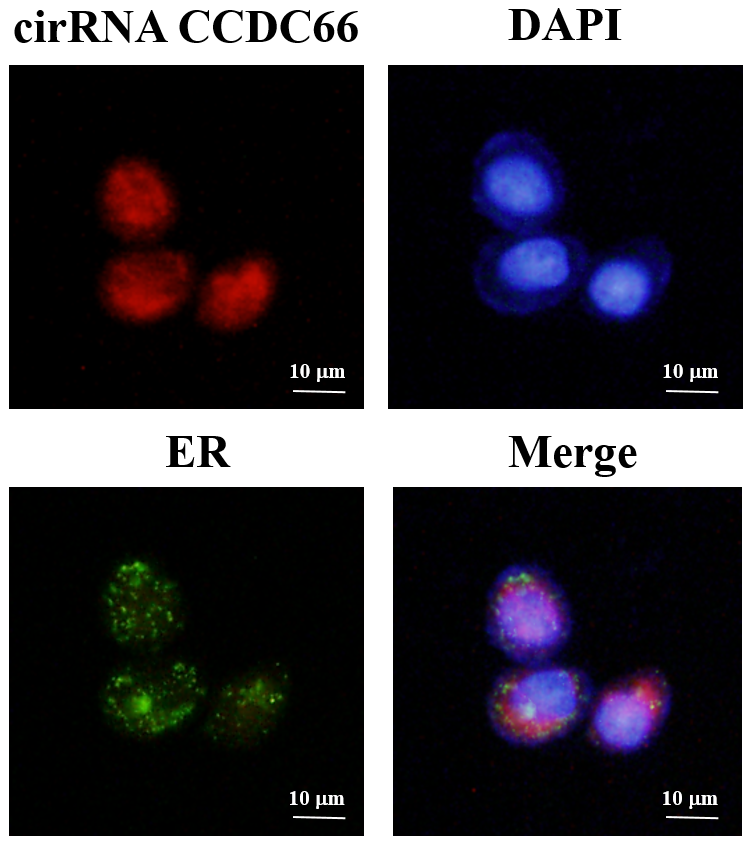


**Additional file 4** Fluorescence immunocytochemical staining of A549 LADC cells. CCDC66 cirRNA was detected by specific probes to cirRNA (biotin-labeled), and then reacting to strptoavidin before visualized by the antibodies to strptoavidin conjugated with Texas red. The endoplasmic reticulum (ER) was labelled by transfecting A549 cells using plasmids encoding ER-targeted Discosoma green fluorescent protein (ER-GFP). Nuclei were stained with fluorescent dye 4', 6-diamidino-2-phenylindole (DAPI, blue fluorescence). A merged image of the above-mentioned markers is shown in the lower right corner. The white bar represents 10 μm. Like SAE2, CCDC66 cirRNA was distributed on the ER and in the cytoplasm.
