# Supplementary material for: The role of HGF-MET pathway and CCDC66 cirRNA expression in EGFR resistance and epithelial-to-mesenchymal transition of lung adenocarcinoma cells
Source: J Hematol Oncol. 2018 May 31;11:74. doi: 10.1186/s13045-018-0557-9 (PMC5984410; doi:10.1186/s13045-018-0557-9)
Supplement: Supplementary file 5 — Prediction of transmembrane and coiled-coil domains in amino acid sequences of TET1 and TET2. (DOCX 335 kb) [file 13045_2018_557_MOESM5_ESM.docx]

## Additional file 5 Prediction of transmembrane and coiled-coil domains in amino acid sequences of TET1 and TET2.

# Prediction of transmembrane and coiled-coil domains in amino acid sequences of TET1 by ProScale (https://web.expasy.org/protscale/)

**TET1**


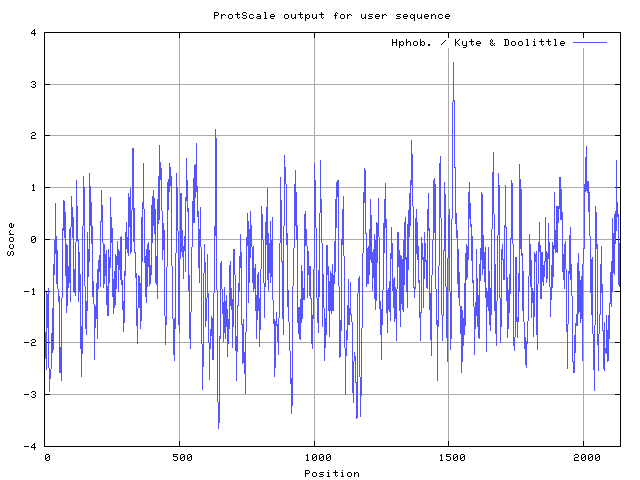


# Results of Subprograms in PSORT II (<http://psort.hgc.jp/form2.html>)

[ALOM: Klein et al's method for TM region allocation](http://psort.hgc.jp/psort/helpwww2.html#ealom)

Init position for calculation: 1

Tentative number of TMS(s) for the threshold 0.5: 1

Number of TMS(s) for threshold 0.5: 1

INTEGRAL Likelihood = -3.19 Transmembrane 1513 -1529

PERIPHERAL Likelihood = 1.91 (at 2006)

ALOM score: -3.19 (number of TMSs: 1)

[MTOP: Prediction of membrane topology (Hartmann et al.)](http://psort.hgc.jp/psort/helpwww2.html#mtop)

Center position for calculation: 1520

Charge difference: -4.0 C(-2.0) - N( 2.0)

N >= C: N-terminal side will be inside

>>> membrane topology: type 2 (cytoplasmic tail 1 to 1513)

[Gavel: prediction of cleavage sites for mitochondrial preseq](http://psort.hgc.jp/psort/helpwww2.html#mit)

R-2 motif at 24 VRK|ED

[COIL: Lupas's algorithm to detect coiled-coil regions](http://psort.hgc.jp/psort/helpwww2.html#lupas)

2059 G 0.71

2060 F 0.81

2061 E 0.99

2062 L 0.99

2063 N 0.99

2064 K 0.99

2065 I 0.99

2066 K 0.99

2067 F 0.99

2068 E 0.99

2069 A 0.99

2070 K 0.99

2071 E 0.99

2072 A 0.99

2073 K 0.99

2074 N 0.99

2075 K 0.99

2076 K 0.99

2077 M 0.99

2078 K 0.99

2079 A 0.99

2080 S 0.99

2081 E 0.99

2082 Q 0.99

2083 K 0.99

2084 D 0.99

2085 Q 0.99

2086 A 0.99

2087 A 0.99

2088 N 0.99

2089 E 0.99

2090 G 0.59

total: 32 residues

[Results of the](http://psort.hgc.jp/psort/helpwww2.html#note)*[k](http://psort.hgc.jp/psort/helpwww2.html#note)*[-NN Prediction](http://psort.hgc.jp/psort/helpwww2.html#note)

*Distribution of this protein could be*

43.5 %: nuclear

17.4 %: cytoplasmic

13.0 %: Golgi

13.0 %: mitochondrial

8.7 %: cytoskeletal

4.3 %: vesicles of secretory system

## SOSUI Result (transmembrane domain predicted by SOSUI web software)

Query title: TET1

Total length: 2136 a.a.

Average of hydrophobicity: -0.653276

### This amino acid sequence is of a MEMBRANE PROTEIN, which has 1 transmembrane helix.

| No. | N terminal | transmembrane region | C terminal | type | length |
| --- | --- | --- | --- | --- | --- |
| 1 | 1506 | TGHHCPTAVMVVLIMVWDGIPLP | 1528 | PRIMARY | 23 |


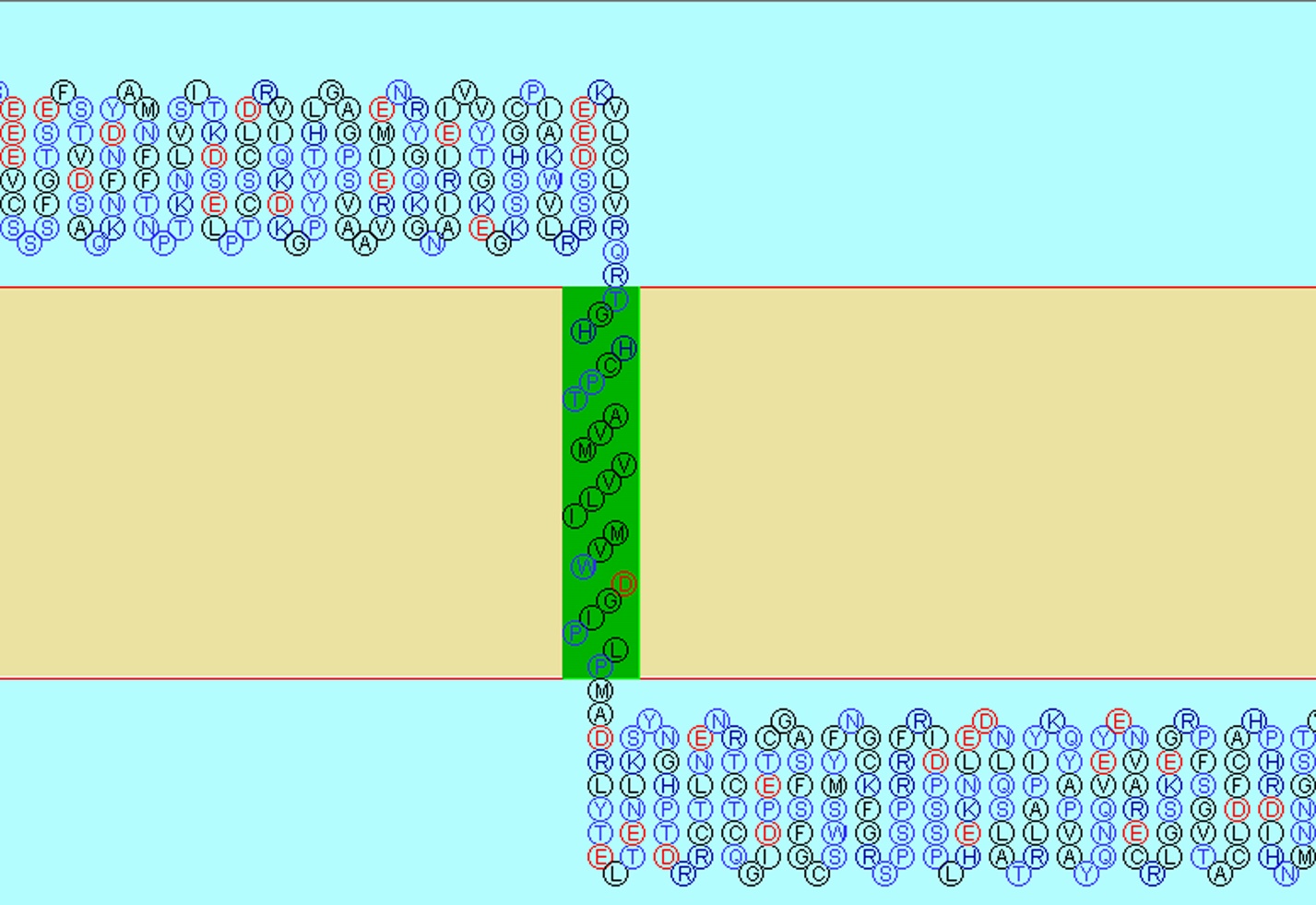


# Prediction of transmembrane and coiled-coil domains in amino acid sequences of TET2

**TET2**


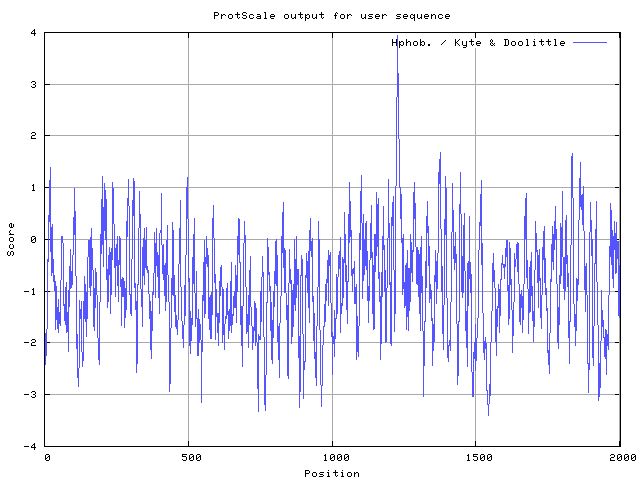


# Results of Subprograms in PSORT II

[ALOM: Klein et al's method for TM region allocation](http://psort.hgc.jp/psort/helpwww2.html#ealom)

Init position for calculation: 1

Tentative number of TMS(s) for the threshold 0.5: 1

Number of TMS(s) for threshold 0.5: 1

INTEGRAL Likelihood = -7.17 Transmembrane 1224 -1240

PERIPHERAL Likelihood = 3.23 (at 1859)

ALOM score: -7.17 (number of TMSs: 1)

[MTOP: Prediction of membrane topology (Hartmann et al.)](http://psort.hgc.jp/psort/helpwww2.html#mtop)

Center position for calculation: 1231

Charge difference: -3.5 C(-2.0) - N( 1.5)

N >= C: N-terminal side will be inside

>>> membrane topology: type 2 (cytoplasmic tail 1 to 1224)

[COIL: Lupas's algorithm to detect coiled-coil regions](http://psort.hgc.jp/psort/helpwww2.html#lupas)

1466 R 0.60

1467 K 0.67

1468 L 0.67

1469 E 0.67

1470 A 0.67

1471 K 0.67

1472 K 0.67

1473 A 0.67

1474 A 0.67

1475 A 0.67

1476 E 0.67

1477 K 0.67

1478 L 0.67

1479 S 0.67

1480 S 0.67

1481 L 0.67

1482 E 0.67

1483 N 0.67

1484 S 0.67

1485 S 0.67

1486 N 0.67

1487 K 0.67

1488 N 0.67

1489 E 0.67

1490 K 0.67

1491 E 0.67

1492 K 0.67

1493 S 0.67

1494 A 0.67

total: 29 residues

**[Results of the](http://psort.hgc.jp/psort/helpwww2.html#note)*[k](http://psort.hgc.jp/psort/helpwww2.html#note)*[-NN Prediction](http://psort.hgc.jp/psort/helpwww2.html#note)**

*Distribution of this protein inside the cell could be*

**33.3 %: Golgi**

**33.3 %: nuclear**

22.2 %: cytoplasmic

11.1 %: mitochondrial

# TMpred output for TET2

**[EMBnet-Server]** Date: Wed Oct 18 7:11:19 2017

Sequence: MEQ...RYI, length: 2002
Prediction parameters: TM-helix length between 17 and 33

## 1.) Possible transmembrane helices

The sequence positions in brackets denominate the core region.
Only scores above 500 are considered significant.

Inside to outside helices : 2 found

from to score center

1221 (1223)1241 (1241) 1426 1231

1280 (1280)1297 (1297) 702 1288

Outside to inside helices : 3 found

from to score center

1223 (1223)1240 (1240) 1317 1231

1280 (1280)1298 (1296) 596 1288

1852 (1855)1876 (1873) 133 1863

## 2.) Table of correspondences

Here is shown, which of the inside->outside helices correspond to which of the outside->inside helices.

Helices shown in brackets are considered insignificant.
A "+"-symbol indicates a preference of this orientation.
A "++"-symbol indicates a strong preference of this orientation.

inside->outside | outside->inside

1221-1241 (21) 1426 + | 1223-1240 (18) 1317

1280-1297 (18) 702 + | 1280-1298 (19) 596

| (1852-1876 (25) 133 ++)

## 3.) Suggested models for transmembrane topology

These suggestions are purely speculative and should be used with **extreme caution** since they are based on the assumption that all transmembrane helices have been found.
In most cases, the Correspondence Table shown above or the prediction plot that is also created should be used for the topology assignment of unknown proteins.

2 possible models considered, only significant TM-segments used

-----> STRONGLY prefered model: N-terminus inside

2 strong transmembrane helices, total score : 2022

# from to length score orientation

1 1221 1241 (21) 1426 i-o

2 1280 1298 (19) 596 o-i

------> alternative model

2 strong transmembrane helices, total score : 2019

# from to length score orientation

1 1223 1240 (18) 1317 o-i

2 1280 1297 (18) 702 i-o


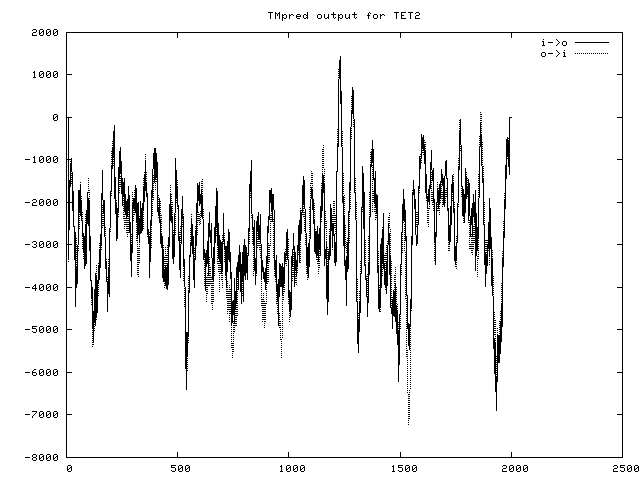


These data indicate that both TET1 and TET2 are transmembrane proteins.
