# Supplementary material for: The role of HGF-MET pathway and CCDC66 cirRNA expression in EGFR resistance and epithelial-to-mesenchymal transition of lung adenocarcinoma cells
Source: J Hematol Oncol. 2018 May 31;11:74. doi: 10.1186/s13045-018-0557-9 (PMC5984410; doi:10.1186/s13045-018-0557-9)
Supplement: Supplementary file 6 — Characterization of monoclonal antibodies to SAE2 and the immunoprecipitated products. (DOCX 807 kb) [file 13045_2018_557_MOESM6_ESM.docx]

**Additional file 6** Characterization of monoclonal antibodies to SAE2 and the immunoprecipitated products.

**A.** Using home-made monoclonal antibodies to precipitate SAE2 form A549 cell lysate, the pull-down 100-kDa protein was detected by silver stain.


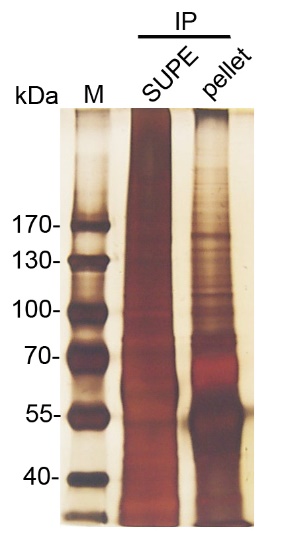


**B** Using home-made monoclonal antibodies to precipitate SAE2 form A549 cell lysate, the pull-down 100-kDa protein was detected immunoblotting using the same home-made monoclonal antibodies to SAE2.


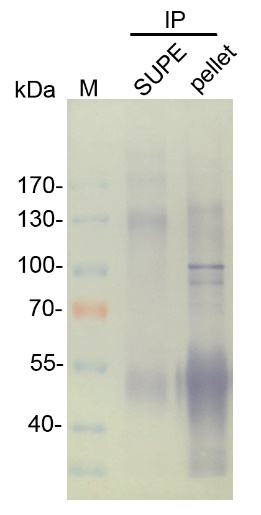


**C. MALDI-TOF analysis of the excised protein band of SAE2**

**
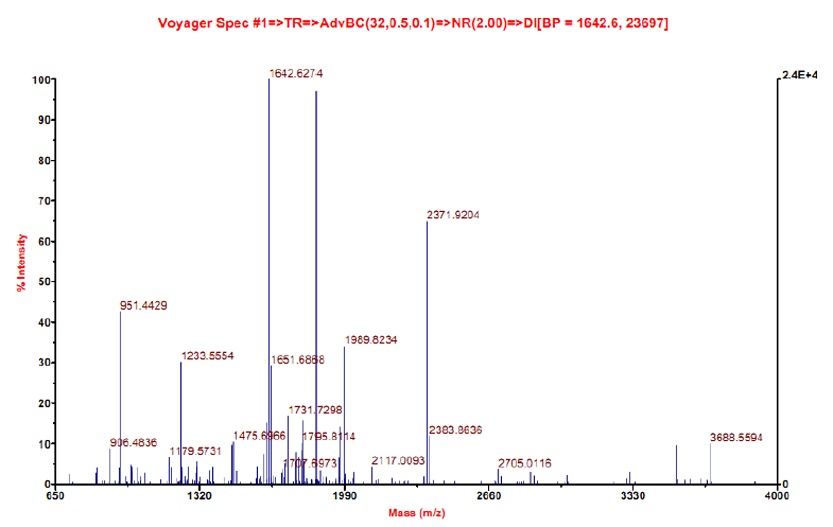
**

**D1.** Protein fragments, which were identified by MALDI-[TOF](http://en.wikipedia.org/wiki/Time-of-flight_mass_spectrometry) analysis, matched to SAE2.  **
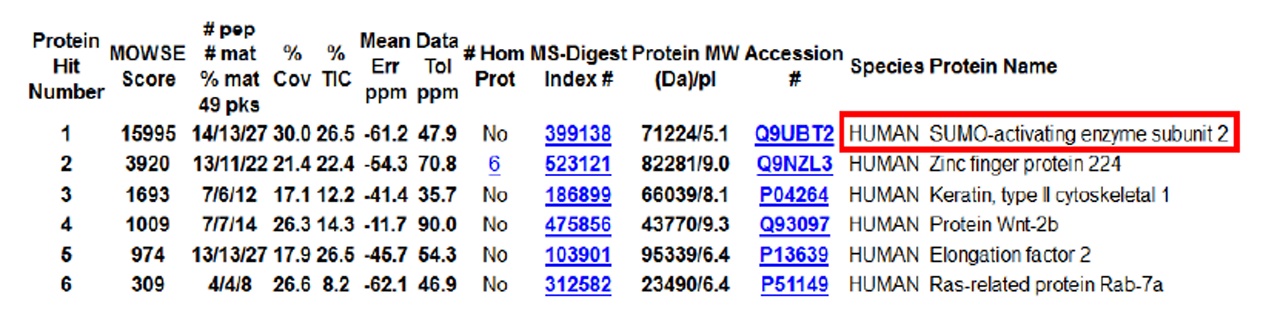
**

**D2.** Protein fragments, which were identified by MALDI-[TOF](http://en.wikipedia.org/wiki/Time-of-flight_mass_spectrometry) analysis, also matched to SAE1, indicating that the 100-kDa protein contained both sequences for SAE2 and SAE1.

**
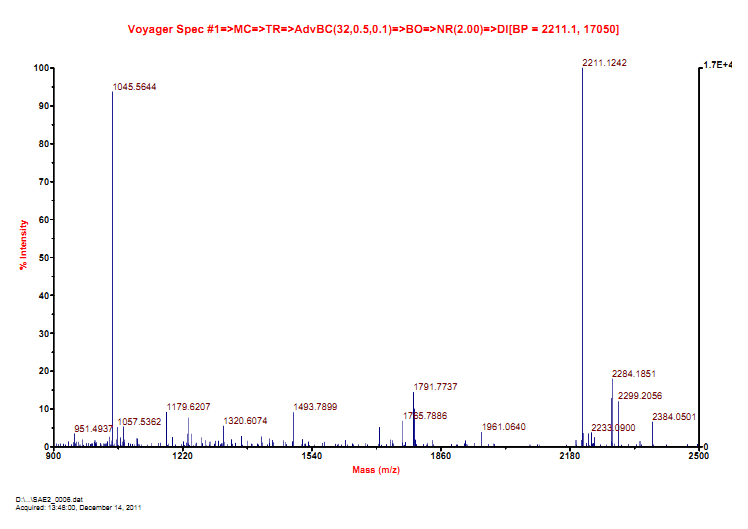
**

**
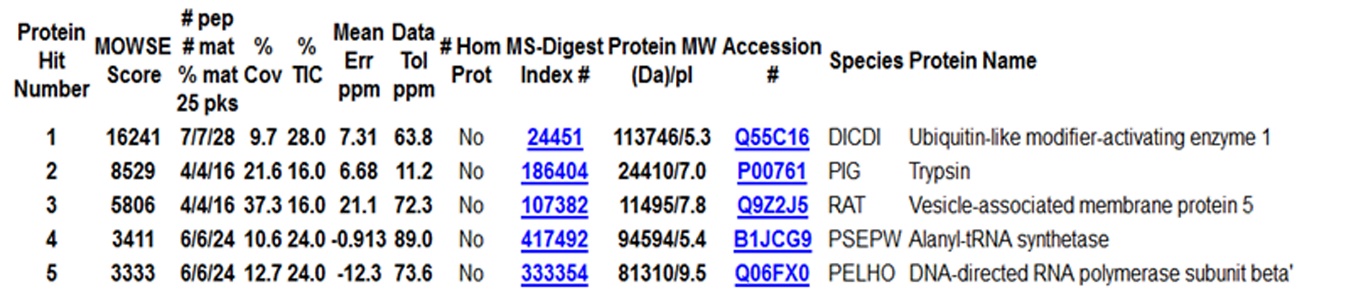
**

**E.** The amino acid fragments recognized MALDI-TOF analysis covered about 30% (192/640 aa’s) of the SAE2 protein, and 33.1% (127/384 aa’s) of the SAE1.

**
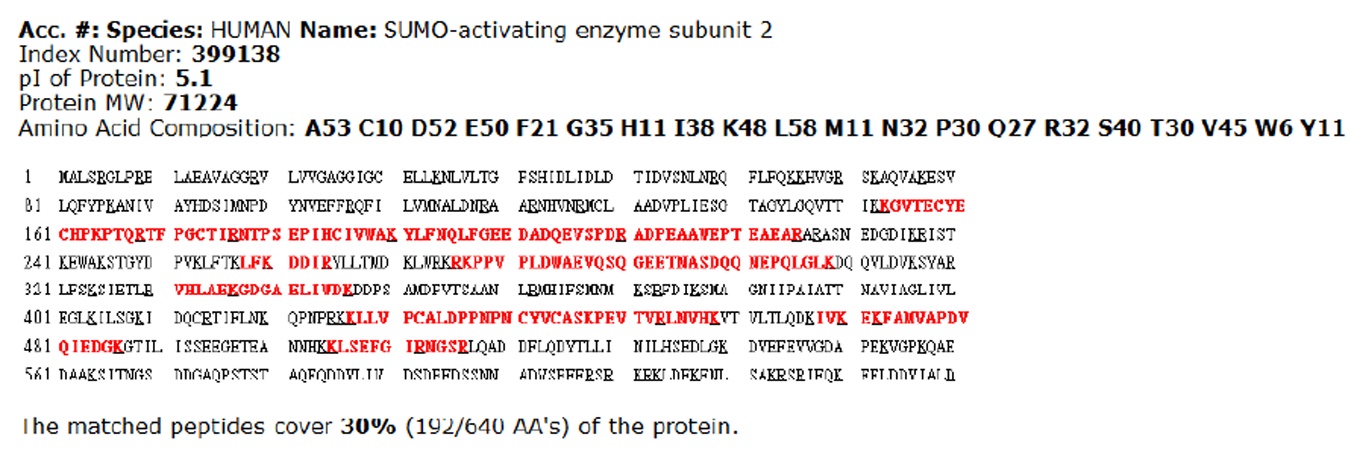
**

**F.** Co-localization of SAE2 and SAE1 on the same 100-kDa protein band as determined by immunoprecipitation and immunoblotting.

**
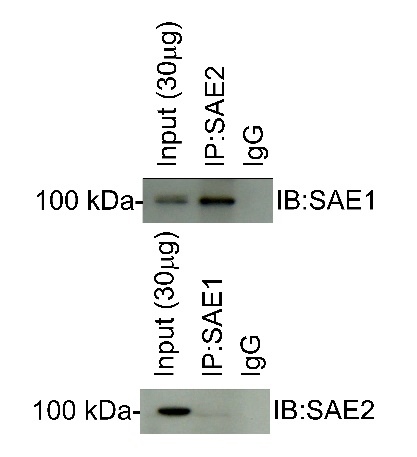
**

**G.** Characterization of the 72-kDa and 100-kDa protein bands that were positively reacting to SAE2 antibodies.

**
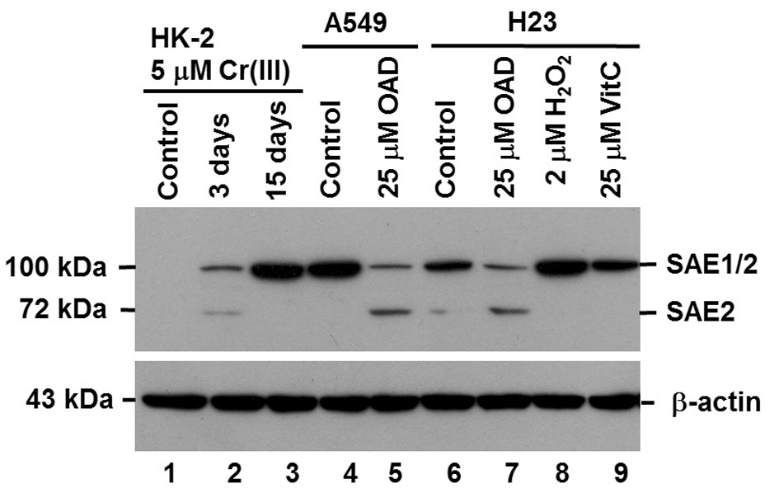
**

**H.** Alignment of amino acid sequences of SAE1 or SAE2 (from human) with those of UBA1 (from Dictyostelium discoideum, in which SAE1 and SAE2 subunits were joint together, not separated).

# H1. Alignment of amino acid sequences between SAE1 and UBA1 by web software LALIGN (https://embnet.vital-it.ch/software/LALIGN_form.html)

Comparison of:

(A) ./wwwtmp/.7768.1.seq SAE1 346 bp - 346 aa

(B) ./wwwtmp/.7768.2.seq UBA1 1017 bp - 1017 aa

35.8% identity in 159 aa overlap (15-172:14-169); score: 299 E(10000): 2.5e-22

20 30 40 50 60 70

SAE1 EAAQYDRQIRLWGLEAQKRLRASRVLLVGLKGLGAEIAKNLILAGVKGLTMLDHEQVTPE

. : :.::. . :..:.. .. ::.:::.::: ::.:.: :::::..:. :.: : .

UBA1 DDALYSRQLYALSHETMKKITSTSVLVVGLQGLGIEIVKDLSLAGVKSVTLYDKELVEIK

20 30 40 50 60 70

80 90 100 110 120 130

SAE1 DPGAQFLIRTGSVGR-NRAEASLERAQNLNPMVDVKVDTEDIEKKPESFFTQFDAVCLTC

: ..:: . .::. .::.: .... .:: . :..:... : . : :. .:..: :.

UBA1 DLSSQFYFSPEQVGKVGRADACFQKVVDLNNY--VRIDVHNGELSDE-FLKKFNVVVLAN

80 90 100 110 120 130

140 150 160 170

SAE1 CSRDVIVKVDQICHKNSIKFFTGDVFGYHGYTFANLGEH

. .::...:: :.:.:.. .. : : : ..::.

UBA1 QPLALQLKVNEFCHANKIHFISVETRGVFGQLFNDFGEQ

140 150 160

**H2**. Alignment of amino acid sequences between SAE2 and UBA1

Comparison of:

(A) ./wwwtmp/.24825.1.seq SAE2 640 bp - 640 aa

(B) ./wwwtmp/.24825.2.seq UBA1 1017 bp - 1017 aa

36.5% identity in 197 aa overlap (9-197:420-616); score: 391 E(10000): 2.6e-31

10 20 30 40 50 60

SAE2 RELAEAVAGGRVLVVGAGGIGCELLKNLVLTGFSH-----IDLIDLDTIDVSNLNRQFLF

. : . . . ..::::.::::.:::... :.. . . :.:::. :::::::::

UBA1 KTLQNKIENLNYFLVGAGAIGCEMLKNFAMMGLGAGPKGLVHVTDMDTIEKSNLNRQFLF

420 430 440 450 460 470

70 80 90 100 110 120

SAE2 QKKHVGRSKAQVAKESVLQFYPKANIVAYHDSI---MNPDYNVEFFRQFILVMNALDNRA

... . . :.:.: ..: . : :. :: . . :: ::: .. : :::::

UBA1 RSSDIQQLKSQTAANAVRVMNPDLNVKAYSLRVGPDTESHYNEEFFNSLDGVCNALDNVE

480 490 500 510 520 530

130 140 150 160 170 180

SAE2 ARNHVNRMCLAADVPLIESGTAGYLGQVTTIKKGVTECYECHPKPTQRTFPGCTIRNTPS

:: ... .:. ::.:::: : :.. .. .:: : : .. .: ::..: :.

UBA1 ARLYMDSQCVYYGKPLLESGTLGTKGNTQVVVPHLTESYSSSRDPPEKGIPVCTLHNFPN

540 550 560 570 580 590

190

SAE2 EPIHCIVWAKYLFNQLF

: : ::. :. ::

UBA1 AIEHTIQWARDTFEGLF

600 610

**Additional file 6** Characterization of monoclonal antibodies to SAE2 and the immunoprecipitated products. SAE2 was immunoprecipitated, and separated by gel electrophoresis. One gel was stained by silver chromogenic reagent. Another gel was stained by immunoblotting. **A**: Using home-made monoclonal antibodies to precipitate SAE2 form A549 cell lysate, the pull-down 100-kDa protein was detected by silver stain, and (**B**) immunoblotting using the same home-made monoclonal antibodies to SAE2. **C**: The protein was resected from the gel and subjected to MALDI-[TOF](http://en.wikipedia.org/wiki/Time-of-flight_mass_spectrometry) analysis. MALDI-[TOF](http://en.wikipedia.org/wiki/Time-of-flight_mass_spectrometry) analysis of the protein fragments. **D**: Protein fragments, which were identified by MALDI-[TOF](http://en.wikipedia.org/wiki/Time-of-flight_mass_spectrometry) analysis, matched to (**D1**) SAE2 and (**D2**) SAE1, indicating that the 100-kDa protein contained both SAE2 and SAE1. **E**: The amino acid fragments recognized MALDI-TOF analysis covered about 30% (192/640 aa’s) of the SAE2 protein, and 33.1% (127/384 aa’s) of the SAE1. **F**: Co-localization of SAE2 and SAE1 on the same 100-kDa protein band as determined by immunoprecipitation and immunoblotting. Cell lysate was immunoprecipitated with antibodies specific SAE2, and then probed with antibodies specific to SAE1 (upper panel); the SAE1 antibodies recognized the SAE2 pull-downed 100-kDa protein. Alternatively, cell lysate was immunoprecipitated with antibodies specific to SAE1, and then the protein-transferred membrane was probed with antibodies specific to SAE2 (lower panel); the SAE2 antibodies recognized the SAE1 pull-downed 100-kDa protein, confirming that both SAE1 and SAE2 were co-localized on the same 100-kDa protein band. **G**: Characterization of the 72-kDa and 100-kDa protein bands that were positively reacting to SAE2 antibodies. Lane 1, control HK-2 cells (immortalized kidney tubular epithelial cells); lane 2, two SAE2-positive proteins, 72-kDa and 100-kDa, were detected following treatment of HK-2 cells with 5 μM trivalent chromium [Cr(III)] for 3 days; lane 3, one 100-kDa SAE2-positive protein band appeared when HK-2 cells were treated with 5 μM Cr(III) for 15 days. Lane 4, A549 cells highly expressed a 100-kDa SAE2. Lane 5, in the presence of 25 μM strong reducing agent, oxalic acid dehydrate (OAD), for 6 hours, two SAE2-positive bands were detected in A549 cell lysates. Lane 6, H23 cells intermediately expressed a 100-kDa SAE2. Lane 7, following treatment with 25 μM of OAD for 6 hours, in H23 cell lysates two SAE2^+^ bands could be detected. Lane 8, following treatment with 2 μM of H_2_O_2_ for 6 hours, protein levels of SAE2 increased evidently in H23 cell lysates. Lane 9, treatment with 25 μM of vitamin C (VitC) did not affect expression patterns or protein levels of SAE2 in H23 cells. These results suggested that expression patterns and protein levels of SAE2 could be regulated by the presence of strong reducing agent or oxidant. The weak reducing agent (VitC) had less effect on SAE2. In an ongoing study, we are determining whether SAE1 and SAE2 subunits are actually joined together by oxidation. **H**: Sequence homology analysis. **H1**: Comparison of amino acid sequences (aa’s) between SAE1 (from human) with those of UBA1 (from Dictyostelium discoideum, in which SAE1 and SAE2 subunits were joint together, not separated). The two sequences contained 35.8% identity in 159 aa overlap. **H2**: Comparison of amino acid sequences between SAE2 with those of UBA1. The two sequences contained 36.5% identity in 197 aa overlap. These data indicated that SAE1 and SAE2 subunits were linked together by a yet to be determined mechanism.
