# Supplementary material for: The role of HGF-MET pathway and CCDC66 cirRNA expression in EGFR resistance and epithelial-to-mesenchymal transition of lung adenocarcinoma cells
Source: J Hematol Oncol. 2018 May 31;11:74. doi: 10.1186/s13045-018-0557-9 (PMC5984410; doi:10.1186/s13045-018-0557-9)
Supplement: Supplementary file 7 — Supplemental methods. (DOCX 20 kb) [file 13045_2018_557_MOESM7_ESM.docx]

**Additional file 7** Supplemental Methods

**Preparation and Characterization of Mouse Antibodies to Human EGFR and SAE2**

DNA sequence corresponding to C-terminal amino acids 2830 to 3780 of EGFR was amplified by primer sequences containing restriction sites respectively. The primer sequences were *EGFR*-f: 5’- CCGTCGACAAATGCTGGGTGCGGAAGAGAAAG-3’ (*XciI* site is underlined) and *EGFR*-r: 5’- GTGCGGCCGCACTAGGGAAAGAAGTCCTGCTG-3’ (*XmaIII* site is underlined).

DNA sequence corresponding to C-terminal amino acids 524 to 1123 of SAE2 was also amplified by primer sequences containing restriction sites. The primer sequences were *SAE2*-f: 5’-TGAGAATTCATGAAAAAGGGTGTGACCGAGTG-3’ (*EcoRI* site is underlined) and *SAE2*-r: 5’- AGCCTCGAGTTATGCAGATGGGTCATCCTTAT-3’ (*XhoI* site is underlined).

The restriction fragment of EGFR or SAE2 was cloned into an expression vector pET-32b^+^ (pET32, Promega KK, Tokyo, Japan). Bacterial colony containing pET32^+^-EGFR or pET32+-SAE2 was selected, and induced by isopropyl-beta-D-thiogalactopyranoside (IPTG) to mass-produce recombinant protein fragments of EGFR or SAE2.

The recombinant protein was purified by a nickel-affinity column. Affinity-purified protein fragments were used to immunize BALB/c mice, and sensitivity of antiserum (OD_405_ > 0.3 at 1:6,000 dilutions) was measured by enzyme-linked immunosorbent assay (ELISA). Specificity of antibodies was determined by the appearance of a 175-kDa band for EGFR or a 100-kDa band for SAE2 in immunoblotting of lung cancer cell extract. Monoclonal antibodies were produced by a hybridoma technique using mouse myeloma cells NS1, and EGFR- or SAE2-specific antibodies were screened by the above-mentioned methods. In order to determine the identity of the 175-kDa and 100-kDa human proteins, the respective bands were excised from Coomassie stained gels and subjected to an analysis of a matrix-assisted laser desorption/ionization and time-of-flight mass spectrometry (MALDI-[TOF](http://en.wikipedia.org/wiki/Time-of-flight_mass_spectrometry)).

The results showed that the 100-kDa protein matched to the SAE2 (Q9UBT2, UniProtKB/Swiss-Prot) and SAE1 (Q55C16) (MS-Fit data were shown in Additional file 6, A-E), suggesting that SAE1 was probably conjugated to SAE2 (As shown in additional file 6, F-H). The matched peptides covered 30.0% (192/640 aa's) of the SAE2, and 33.1% (127/384 aa’s) of the SAE1.

**Immunoprecipitation, Gel Electrophoresis and Protein Analysis by MALDI-TOF**

The routines procedures in the lab were followed for immunoprecipitation, gel electrophoresis and protein analysis by MALDI-TOF.^1-5^ Total cell lysate was prepared by mixing 5 × 10^7^ cells/100 μL phosphate-buffered saline with equal volume of 2 × NP-40 lysis buffer [40 mM Tris-HCl, pH 7.6, 2 mM EDTA, 300 mM NaCl, 2 mM phenylmethylsulfonylfluoride (PMSF)] , and 2% NP-40. Protein G sepharose^TM^ (Amersham Biosciences AB, Uppsala, Sweden) was pre-washed before mixing with 500 μg of total cell lysate. The reaction mixture was incubated at 4°C for 2 hr, and then centrifuged at 800 × g for 1 min. The supernatant was reacted with 5 μg of purified monoclonal antibodies and 20 μl of freshly prepared protein G sepharose at 4°C for 18 hr. The reaction mixture was centrifuged at 800 × g for 1 min. After removal of the supernatant, the precipitate was washed with 1 × PBS, and dissolved into loading buffer (50 mM Tris, pH 6.8, 150 mM NaCl, 1 mM disodium EDTA, 1 mM PMSF, 10% glycerol, 5% β-mercaptoethanol, 1% SDS and 0.01% bromophenol blue). Eletrophoresis was carried out in two 10% polyacrylamide gels with 4.5% stacking. One gel was processed for immunoblotting,^2^ and the other gel was stained with Coomasie blue. Proteins on the gel, which corresponded to the immunopositive bands, were extracted from the gel for identity analysis by MALDI-TOF on a Voyager-DE^TM^ pro biospectrometry workstation (Applied Biosystems, Milpitas, CA, USA). Fragments of peptide fingerprints were matched with those on the SwissProt database by MS-fit (ProteinProspector 4.0.5., The Regents of the University of California). After electrophoresis, proteins on the first gel were transferred to a nitrocellulose membrane for immunoblotting. The membrane was probed with specific antibodies. The signal was amplified by biotin-labelled goat anti-mouse IgG, and peroxidase-conjugated streptavidin. The protein was visualized by exposing the membrane to an X-Omat film (Eastman Kodak, Rochester, NY) with enhanced chemiluminescent reagent (NEN, Boston, MA).

**Lentivirus Production and Infection**

Lentiviral vector carrying shRNA was prepared by a three-plasmid transfection method. Briefly, the pMD.G containing vesicular stomatitis virus glycoprotein, and pCMV-Δ 8.91carrying HIV-based packaging plasmid were co-transfected with the shRNA-expressing lentiviral vector into 293T cells.^6^ The medium was changed to fresh DMEM containing 10 mg/mL BSA 24 hours post-transfection. The supernatant containing lentivirus was collected at 48 hour and 72 hour post-transfection, respectively, and was used to infect target cells in the presence of 8 μg/ml polybrene. After infection, the cells were selected by 1 μg/ml puromycin.

**References**

1. Chen CY, Fang HY, Chiou SH, Yi SE, Huang CY, Chiang SF, et al. [Sumoylation of eukaryotic elongation factor 2 is vital for protein stability and anti-apoptotic activity in lung adenocarcinoma cells.](http://www.ncbi.nlm.nih.gov/pubmed/21554491) Cancer Sci. 2011;102:1582-1589.
2. [Chen JT](http://www.ncbi.nlm.nih.gov/entrez/query.fcgi?db=pubmed&cmd=Search&term=%22Chen+JT%22%5BAuthor%5D), [Lin TS](http://www.ncbi.nlm.nih.gov/entrez/query.fcgi?db=pubmed&cmd=Search&term=%22Lin+TS%22%5BAuthor%5D), [Chow KC](http://www.ncbi.nlm.nih.gov/entrez/query.fcgi?db=pubmed&cmd=Search&term=%22Chow+KC%22%5BAuthor%5D), Huang HH, Chiou SH, Chiang SF, et al. Cigarette smoking induces overexpression of HGF in type II pneumocytes and lung cancer cells. [Am J Respir Cell Mol Biol](javascript:AL_get(this,%20'jour',%20'Am%20J%20Respir%20Cell%20Mol%20Biol.');). 2006;34:264-273.
3. Chiang YY, Chen SL, Hsiao YT, Huang CH, Lin TY, Chiang IP, et al. [Nuclear expression of dynamin-related protein 1 in lung adenocarcinomas.](http://www.ncbi.nlm.nih.gov/pubmed/19525928) Mod Pathol. 2009;22:1139-1150.
4. [Fang HY](http://www.ncbi.nlm.nih.gov/pubmed?term=Fang%20HY%5BAuthor%5D&cauthor=true&cauthor_uid=20332122), [Chang CL](http://www.ncbi.nlm.nih.gov/pubmed?term=Chang%20CL%5BAuthor%5D&cauthor=true&cauthor_uid=20332122), [Hsu SH](http://www.ncbi.nlm.nih.gov/pubmed?term=Hsu%20SH%5BAuthor%5D&cauthor=true&cauthor_uid=20332122), Huang CY, Chiang SF, Chiou SH, et al. ATPase family AAA domain-containing 3A is a novel anti-apoptotic factor in lung adenocarcinoma cells. [J Cell Sci](http://www.ncbi.nlm.nih.gov/pubmed/20332122). 2010;123:1171-1180.
5. Hsu NY, Ho HC, Chow KC, Lin TY, Shih CS, Wang LS, et al. [Overexpression of dihydrodiol dehydrogenase as a prognostic marker of non-small cell lung cancer.](http://www.ncbi.nlm.nih.gov/pubmed/11289154) Cancer Res. 2001;61:2727-2731.
6. Smith JS, [Alderete B](http://www.ncbi.nlm.nih.gov/pubmed?term=Alderete%20B%5BAuthor%5D&cauthor=true&cauthor_uid=10435596), [Minn Y](http://www.ncbi.nlm.nih.gov/pubmed?term=Minn%20Y%5BAuthor%5D&cauthor=true&cauthor_uid=10435596),  [Borell TJ](http://www.ncbi.nlm.nih.gov/pubmed/?term=Borell%20TJ%5BAuthor%5D&cauthor=true&cauthor_uid=10435596), [Perry A](http://www.ncbi.nlm.nih.gov/pubmed/?term=Perry%20A%5BAuthor%5D&cauthor=true&cauthor_uid=10435596), [Mohapatra G](http://www.ncbi.nlm.nih.gov/pubmed/?term=Mohapatra%20G%5BAuthor%5D&cauthor=true&cauthor_uid=10435596), et al. Localization of common deletion regions on 1p and 19q in human glioma and their association with histological subtype. Oncogene. 1999;18:4144-4152.
